# CSF biomarkers are differentially linked to brain areas high and low in noradrenaline, dopamine and serotonin across the Alzheimer’s disease spectrum

**DOI:** 10.1093/braincomms/fcaf031

**Published:** 2025-01-23

**Authors:** Lena Haag, Elisa Lancini, Renat Yakupov, Gabriel Ziegler, Yeo-Jin Yi, Falk Lüsebrink, Wenzel Glanz, Oliver Peters, Eike Jakob Spruth, Slawek Altenstein, Josef Priller, Luisa Sophie Schneider, Xiao Wang, Lukas Preis, Frederic Brosseron, Nina Roy-Kluth, Klaus Fliessbach, Michael Wagner, Steffen Wolfsgruber, Luca Kleineidam, Alfredo Ramirez, Annika Spottke, Frank Jessen, Jens Wiltfang, Anja Schneider, Niels Hansen, Ayda Rostamzadeh, Katharina Buerger, Michael Ewers, Robert Perneczky, Daniel Janowitz, Boris-Stephan Rauchmann, Stefan Teipel, Ingo Kilimann, Doreen Goerss, Christoph Laske, Matthias H Munk, Michael Heneka, Peter Dechent, Stefan Hetzer, Klaus Scheffler, Emrah Düzel, Matthew J Betts, Dorothea Hämmerer

**Affiliations:** Institute of Cognitive Neurology and Dementia Research (IKND), 39120 Magdeburg, Germany; German Center for Neurodegenerative Diseases (DZNE), 39120 Magdeburg, Germany; Institute of Cognitive Neurology and Dementia Research (IKND), 39120 Magdeburg, Germany; German Center for Neurodegenerative Diseases (DZNE), 39120 Magdeburg, Germany; Institute of Cognitive Neurology and Dementia Research (IKND), 39120 Magdeburg, Germany; German Center for Neurodegenerative Diseases (DZNE), 39120 Magdeburg, Germany; Institute of Cognitive Neurology and Dementia Research (IKND), 39120 Magdeburg, Germany; German Center for Neurodegenerative Diseases (DZNE), 39120 Magdeburg, Germany; Institute of Cognitive Neurology and Dementia Research (IKND), 39120 Magdeburg, Germany; German Center for Neurodegenerative Diseases (DZNE), 39120 Magdeburg, Germany; Institute of Cognitive Neurology and Dementia Research (IKND), 39120 Magdeburg, Germany; German Center for Neurodegenerative Diseases (DZNE), 39120 Magdeburg, Germany; German Center for Neurodegenerative Diseases (DZNE), 10117 Berlin, Germany; Department of Psychiatry and Psychotherapy, Charité—Universitätsmedizin Berlin, corporate member of Freie Universität Berlin, Humboldt—Universität zu Berlin, 12200 Berlin, Germany; German Center for Neurodegenerative Diseases (DZNE), 10117 Berlin, Germany; Department of Psychiatry and Psychotherapy, Charité, Humboldt University Berlin, 10117 Berlin, Germany; German Center for Neurodegenerative Diseases (DZNE), 10117 Berlin, Germany; Department of Psychiatry and Psychotherapy, Charité, Humboldt University Berlin, 10117 Berlin, Germany; German Center for Neurodegenerative Diseases (DZNE), 10117 Berlin, Germany; Department of Psychiatry and Psychotherapy, Charité, Humboldt University Berlin, 10117 Berlin, Germany; Department of Psychiatry and Psychotherapy, School of Medicine, Technical University of Munich, 81675 Munich, Germany; UK Dementia Research Institute, University of Edinburgh, Edinburgh EH16 4SB, UK; Department of Psychiatry and Psychotherapy, Charité—Universitätsmedizin Berlin, corporate member of Freie Universität Berlin, Humboldt—Universität zu Berlin, 12200 Berlin, Germany; Department of Psychiatry and Psychotherapy, Charité—Universitätsmedizin Berlin, corporate member of Freie Universität Berlin, Humboldt—Universität zu Berlin, 12200 Berlin, Germany; Department of Psychiatry and Psychotherapy, Charité—Universitätsmedizin Berlin, corporate member of Freie Universität Berlin, Humboldt—Universität zu Berlin, 12200 Berlin, Germany; German Center for Neurodegenerative Diseases (DZNE), Bonn, 53127 Bonn, Germany; German Center for Neurodegenerative Diseases (DZNE), Bonn, 53127 Bonn, Germany; German Center for Neurodegenerative Diseases (DZNE), Bonn, 53127 Bonn, Germany; Deptartment of Neurodegenerative Disease and Geriatric Psychiatry/Psychiatry, University of Bonn Medical Center, 53127 Bonn, Germany; German Center for Neurodegenerative Diseases (DZNE), Bonn, 53127 Bonn, Germany; Deptartment of Neurodegenerative Disease and Geriatric Psychiatry/Psychiatry, University of Bonn Medical Center, 53127 Bonn, Germany; German Center for Neurodegenerative Diseases (DZNE), Bonn, 53127 Bonn, Germany; Deptartment of Neurodegenerative Disease and Geriatric Psychiatry/Psychiatry, University of Bonn Medical Center, 53127 Bonn, Germany; German Center for Neurodegenerative Diseases (DZNE), Bonn, 53127 Bonn, Germany; Deptartment of Neurodegenerative Disease and Geriatric Psychiatry/Psychiatry, University of Bonn Medical Center, 53127 Bonn, Germany; German Center for Neurodegenerative Diseases (DZNE), Bonn, 53127 Bonn, Germany; Deptartment of Neurodegenerative Disease and Geriatric Psychiatry/Psychiatry, University of Bonn Medical Center, 53127 Bonn, Germany; Excellence Cluster on Cellular Stress Responses in Aging-Associated Diseases (CECAD), University of Cologne, 50931 Cologne, Germany; Division of Neurogenetics and Molecular Psychiatry, Department of Psychiatry and Psychotherapy, Faculty of Medicine and University Hospital Cologne, University of Cologne, 50931 Cologne, Germany; Department of Psychiatry & Glenn Biggs Institute for Alzheimer's and Neurodegenerative Diseases, University of Texas Health Science Center, San Antonio, TX 78229, USA; German Center for Neurodegenerative Diseases (DZNE), Bonn, 53127 Bonn, Germany; Department of Neurology, University of Bonn, 53127 Bonn, Germany; Excellence Cluster on Cellular Stress Responses in Aging-Associated Diseases (CECAD), University of Cologne, 50931 Cologne, Germany; German Center for Neurodegenerative Diseases (DZNE), 37075 Goettingen, Germany; Department of Psychiatry, University of Cologne, Medical Faculty, 50924 Cologne, Germany; German Center for Neurodegenerative Diseases (DZNE), 37075 Goettingen, Germany; Department of Psychiatry and Psychotherapy, University Medical Center Goettingen, University of Goettingen, 37075 Goettingen, Germany; Neurosciences and Signaling Group, Institute of Biomedicine (iBiMED), Department of Medical Sciences, University of Aveiro, 3810-198 Aveiro, Portugal; Department of Psychiatry and Psychotherapy, University Medical Center Goettingen, University of Goettingen, 37075 Goettingen, Germany; Department of Psychiatry and Psychotherapy, University Medical Center Goettingen, University of Goettingen, 37075 Goettingen, Germany; Department of Psychiatry, University of Cologne, Medical Faculty, 50924 Cologne, Germany; German Center for Neurodegenerative Diseases (DZNE), 81377 Munich, Germany; Institute for Stroke and Dementia Research (ISD), University Hospital, LMU Munich, 81377 Munich, Germany; German Center for Neurodegenerative Diseases (DZNE), 81377 Munich, Germany; Institute for Stroke and Dementia Research (ISD), University Hospital, LMU Munich, 81377 Munich, Germany; German Center for Neurodegenerative Diseases (DZNE), 81377 Munich, Germany; Department of Psychiatry and Psychotherapy, University Hospital, LMU Munich, 80336 Munich, Germany; Munich Cluster for Systems Neurology (SyNergy), 81377 Munich, Germany; Ageing Epidemiology Research Unit (AGE), School of Public Health, Imperial College London, London W12 0BZ, UK; Institute for Stroke and Dementia Research (ISD), University Hospital, LMU Munich, 81377 Munich, Germany; Department of Psychiatry and Psychotherapy, University Hospital, LMU Munich, 80336 Munich, Germany; Sheffield Institute for Translational Neuroscience (SITraN), University of Sheffield, Sheffield S10 2HQ, UK; Department of Neuroradiology, University Hospital LMU, 81377 Munich, Germany; German Center for Neurodegenerative Diseases (DZNE), 18147 Rostock, Germany; Department of Psychosomatic Medicine, Rostock University Medical Center, 18147 Rostock, Germany; German Center for Neurodegenerative Diseases (DZNE), 18147 Rostock, Germany; Department of Psychosomatic Medicine, Rostock University Medical Center, 18147 Rostock, Germany; German Center for Neurodegenerative Diseases (DZNE), 18147 Rostock, Germany; Department of Psychosomatic Medicine, Rostock University Medical Center, 18147 Rostock, Germany; German Center for Neurodegenerative Diseases (DZNE), 72076 Tübingen, Germany; Section for Dementia Research, Hertie Institute for Clinical Brain Research and Department of Psychiatry and Psychotherapy, University of Tübingen, 72076 Tübingen, Germany; German Center for Neurodegenerative Diseases (DZNE), 72076 Tübingen, Germany; Department of Psychiatry and Psychotherapy, University of Tübingen, 72076 Tübingen, Germany; Luxembourg Centre for Systems Biomedicine (LCSB), University of Luxembourg, L-4367 Belvaux, Luxembourg; MR-Research in Neurosciences, Department of Cognitive Neurology, Georg-August-University Goettingen, 37075 Goettingen, Germany; Berlin Center for Advanced Neuroimaging, Charité—Universitätsmedizin Berlin, 10117 Berlin, Germany; Department for Biomedical Magnetic Resonance, University of Tübingen, 72076 Tübingen, Germany; Institute of Cognitive Neurology and Dementia Research (IKND), 39120 Magdeburg, Germany; German Center for Neurodegenerative Diseases (DZNE), 39120 Magdeburg, Germany; Institute of Cognitive Neurology and Dementia Research (IKND), 39120 Magdeburg, Germany; German Center for Neurodegenerative Diseases (DZNE), 39120 Magdeburg, Germany; Center for Behavioral Brain Sciences, University of Magdeburg, 39120 Magdeburg, Germany; Institute of Cognitive Neurology and Dementia Research (IKND), 39120 Magdeburg, Germany; German Center for Neurodegenerative Diseases (DZNE), 39120 Magdeburg, Germany; Center for Behavioral Brain Sciences, University of Magdeburg, 39120 Magdeburg, Germany; Department of Psychology, University of Innsbruck, 6020 Innsbruck, Austria; Institute of Cognitive Neuroscience, University College London, London WC1E 6BT, UK

**Keywords:** p-tau, amyloid-ß, subjective cognitive decline, Alzheimer’s disease, structural equation modelling

## Abstract

Neurotransmitter systems of noradrenaline, dopamine, serotonin and acetylcholine are implicated in cognitive functions such as memory, learning and attention and are known to be altered in neurodegenerative diseases like Alzheimer’s disease. Specific brain structures involved in these systems, e.g. the locus coeruleus, the main source of noradrenaline in the cortex, are in fact affected earliest by Alzheimer’s disease tau pathology. Preserved volumetric neurotransmitter specific brain areas could therefore be an important neural resource for cognitive reserve in aging. The aim of this study was to determine whether volumes of brain areas known to be high in neurotransmitter receptors are relatively preserved in individuals with lower levels of Alzheimer’s disease pathology. Based on the Human Protein Atlas for neurotransmitter receptor distribution, we distinguished between ‘areas high and low’ in noradrenaline, dopamine, serotonin and acetylcholine and assessed associations of atrophy in those areas with CSF amyloid-ß 42/40, CSF phosphorylated tau protein and cognitive function across healthy controls (*n* = 122), individuals with subjective cognitive decline (*n* = 156), mild cognitive impairment or mild Alzheimer’s disease dementia (*n* = 126) using structural equation modelling. CSF pathology markers were inversely correlated and showed a stronger association with disease severity, suggesting distinguishable interrelatedness of these biomarkers depending on the stage of Alzheimer’s disease dementia. Across groups, amyloid pathology was linked to atrophy in areas high as well as low in neurotransmitter receptor densities, while tau pathology did not show any significant link to brain area volumes for any of the neurotransmitters. Within disease severity groups, individuals with more amyloid pathology showed more atrophy only in ‘areas high in noradrenaline’, whereas for dopamine tau pathology was linked to higher volumes in areas low in receptor density possibly indicating compensatory mechanisms. Furthermore, individuals with more tau pathology showed a selective decrease in memory function while amyloid pathology was related to a decline in executive function and language capacity as well as memory function. In summary, our analyses highlight the benefits of investigating disease-relevant factors in Alzheimer’s disease using a multivariate multigroup approach. Assessing multivariate dependencies in different disease stages and across individuals revealed selective links of pathologies, cognitive decline and atrophy in particular for areas modulated by noradrenaline, dopamine and serotonin.

## Teaser

Haag *et al*. investigated specific neurotransmitter systems in Alzheimer’s disease separating brain areas into high versus low noradrenergic, dopaminergic, serotonergic and cholinergic receptor density areas. Amyloid pathology was linked to worse cognitive function—specifically memory, language and executive function—as well as to atrophy in noradrenergic high areas.

## Graphical Abstract

**graphical abstract:**
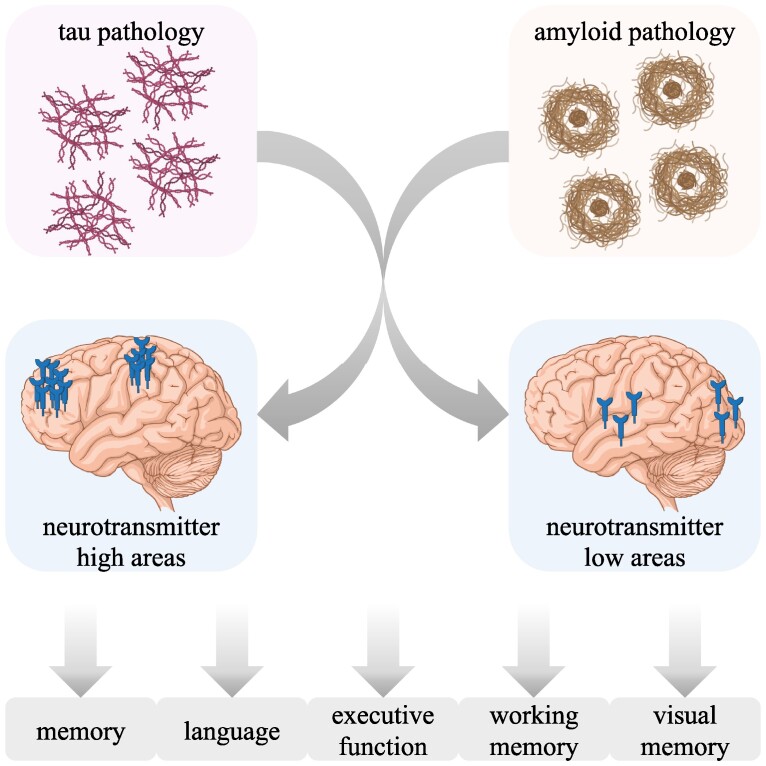

## Introduction

Alzheimer’s disease is the most common type of dementia worldwide^1^ and is characterized by memory impairment and presence of elevated aggregated amyloid-ß (Aß) and pathological phosphorylated tau protein (p-tau) in neurons.^2^ Preclinical and prodromal stages of Alzheimer’s disease include subjective cognitive decline (SCD),^3,4^ which is characterized by self-experienced cognitive decline that does not reach the level of objective impairment required for the clinical diagnosis of mild cognitive impairment (MCI), and MCI, characterized by objective cognitive decline that does not yet fulfil the criteria for a diagnosis of dementia.^5^ Individuals included in these groups are at higher risk of developing Alzheimer’s disease dementia at later stages.^6,7^

Typical aging and early stages of Alzheimer’s disease are characterized by neuronal loss and accumulation of neurofibrillary tau protein tangles in the locus coeruleus (LC), which is the main source of noradrenaline (NA) in the brain.^8^ In fact, LC neuronal loss in Alzheimer’s disease is higher compared to other subcortical nuclei, such as the cholinergic nucleus basalis of Meynert (NBM) and the dopaminergic substantia nigra (SN) pars compacta.^9^ Interestingly, a more preserved LC-NA system appears to increase cognitive reserve^10^ and is associated with changes in memory and cognitive decline^11,12^ in aging and Alzheimer’s disease.^9,13^ Nevertheless, a vulnerability of other neurotransmitter systems such as the dopaminergic (DA), serotonergic (HT) and cholinergic (ACh) system is also implicated in cognitive decline in normal aging and also in early neurodegenerative stages.^14^ For instance, binding potential loss indicative of reduced dopamine transporters in the striatum, hippocampus and caudate nucleus is already observed in the predementia stage of MCI.^15^ A post-mortem study in AD patients observed a decrease of serotonin synthesis in the raphe nuclei.^16^ Mieling *et al*.^17^ could recently show that atrophy in the cholinergic NBM could be used as an imaging biomarker for diagnosis of Alzheimer’s disease, suggesting relevant changes in this neurotransmitter system in the progression of the disease. Since adrenoceptor and transporter densities of neurotransmitters differ regionally as known from earlier post-mortem studies,^18,19^*in vivo* analyses of selective atrophy in specific neurotransmitter target areas as well as links of atrophies to pathology levels and cognitive decline can contribute to our understanding of neurotransmitter-related factors supporting neural reserve in aging and neurodegeneration.

In this paper, we aim to investigate the relevance of different neurotransmitter systems in Alzheimer’s disease by relating interindividual differences in regional brain volumes of projection areas with high versus low neurotransmitter dependencies—as evident in receptor densities—to disease biomarkers (CSF p-tau and Aß) as well as cognitive performance in a cross-sectional sample of three participant groups: (i) healthy controls (HCs), (ii) SCD and (iii) MCI/mild Alzheimer’s disease dementia patients (AD). In doing so, we will control for the influence of risk factors for Alzheimer’s disease (age,^20,21^ female gender,^22,23^ low education level,^24-26^*ApoE4*-positive carrier status,^27,28^ vascular lesions^29^) on this relationship. These analyses will be carried out with structural equation modelling (SEM), a multivariate statistical approach, which allows to test for interrelations of several variables based on a model of their mutual influence, while testing for group differences in model properties.^30^ If a decline in neurotransmission indeed provides an important contribution to cognitive and physiological reserve in Alzheimer’s disease, we assume that in particular atrophies in areas which are high (as compared to low) in receptor densities are related to interindividual differences in disease markers and cognition.

### Hypotheses

We focus on the following research hypotheses:

- Interindividual differences in atrophy of brain volumes in healthy aging, preclinical and clinical Alzheimer’s disease differ for brain regions that are high or low in specific neurotransmitter receptors, pointing to different disease trajectories of brain areas high and low in NA, DA, HT or ACh.
- CSF biomarkers of tau and amyloid pathologies are differentially related to areas high and low in the specific neurotransmitters with a stronger association with atrophy in high areas.
- Cognitive decline shows a stronger association with atrophy in high areas.

## Materials and methods

### Participants

**Figure 1:**
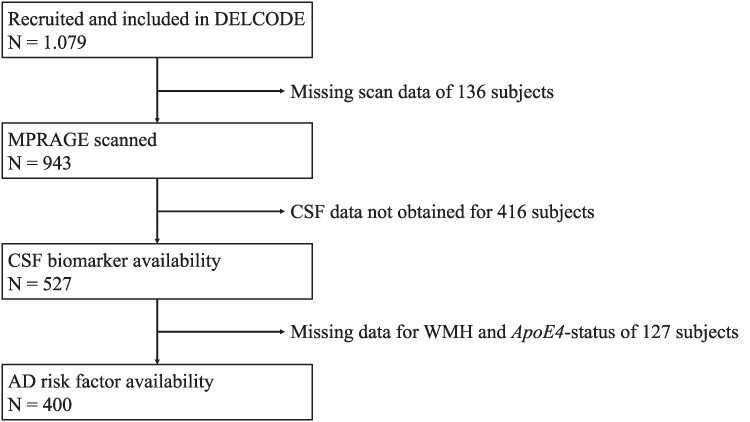
Flowchart on participant selection from the DELCODE study.

A total of 400 participants were included. One thousand and seventy-nine participants were initially screened, which are part of the multicentric DZNE-Longitudinal Cognitive Impairment and Dementia Study (DELCODE), carried out across 11 different study sites in DZNE institutes in Germany.^31^ Written informed consent was obtained from all participants before inclusion, and the study protocol was approved by all local institutional ethical committees. Main exclusion criteria were major depressive or psychiatric disorders, any form of dementia other than Alzheimer’s disease dementia, as well as intake of psychoactive or anti-dementia treatment. Participants were aged between 59 and 89 years, and data were collected between 2014 and 2017. Five hundred fifty men and 529 women were recruited, and participants were initially grouped into HCs (*n* = 236), first-degree relatives to Alzheimer’s patients (*n* = 82), SCD (*n* = 444), amnestic MCI (*n* = 191) and mild Alzheimer’s disease dementia (*n* = 126) by psychiatric and neurological examination and neuropsychological assessment by experienced study physicians. SCD was diagnosed if participants reported self-perceived cognitive decline and if their neuropsychological test score was up to −1.5 SD lower as compared to an age, gender and educational typical value based on the Consortium to Establish a Registry for Alzheimer’s Disease (CERAD). Amnestic MCI was defined as lower than a SD of −1.5 in the delayed recall trial of the CERAD word-list episodic memory tests. Individuals with mild Alzheimer’s disease dementia scored ≥18 points in the Mini Mental State Examination (MMSE).^3,31-33^ The total sample size included in our investigations was *n* = 400 due to optional CSF collection by additional lumbar puncture for biomarker status and availability of additional demographic and clinical data (Fig. 1). Therefore, subgroups were merged to a healthy (HC and first-degree relatives) (*n* = 122), preclinical (SCD) (*n* = 152) and clinical group (amnestic MCI and mild dementia due to Alzheimer’s disease) (*n* = 126).

General demographic and clinical data included age, gender, nationality, height, weight, body mass index, medical history, medication and physical and neurological examination findings. Further clinical measures included were the MMSE, Clinical Dementia Rating (CDR), Geriatric Depression Scale (GDS), Geriatric Anxiety Inventory (GAI), Neuropsychiatric Inventory (NPI-Q) and Functional Activities Questionnaire (FAQ). The level of education was assessed by questionnaire and classified into eight school levels with higher values indicating more advanced education. Neuropsychological testing was performed by a DELCODE test battery (DELCODE-NP) with amongst others Everyday Cognition questionnaire (ECog), Alzheimer’s Disease Assessment Scale—Cognitive Subscale (ADAScog13), Free and Cued Selective Reminding Test with Immediate Recall (FCSRT-IR), Symbol-Digit-Modalities Test (SDMT), Wechsler Memory Scale (WSM-R) and the computer-based Face Name Associative Recognition Test (FNART).

### MRI and biomarker data acquisition

Brain volume was assessed with a T_1_-weighted magnetization prepared rapid gradient echo on 3T (TR = 2500 ms, TE = 4.33 ms, TI = 1100 ms, 256 × 256 mm^2^ FOV, 192 sagittal slices, isotropic 1 mm^3^ voxel size, 5:08 acquisition time), fast low angle shot (TR = 20 ms, TE = 5.56 ms, 320 × 320 mm^2^ FOV, 192 axial slices, isotropic 0.75 mm^3^ voxel size, 13:50 acquisition time) and flow-attenuated inversion recovery (FLAIR) (TR = 5000 ms, TE = 394 ms, TI = 1800 ms, 256 × 256 mm^2^ FOV, 192 sagittal slices, isotropic 1 mm^3^ voxel size, 7:02 acquisition time) sequences.

Data of brain biomarkers such as amyloid-ß 38, 40 and 42 and total and phosphorylated tau protein 181 were available from CSF analyses (*n* = 527), and a ratio was calculated for Aß42/40. *ApoE4* carrier status was assessed in blood samples by real-time polymerase chain reaction of single nucleotide polymorphisms.^31^

### Data analyses

Cognitive variables were estimated as latent variables as reported by an earlier SEM analysis study,^34-36^ which were based on learning and memory tests included in the extensive DELCODE-NP.

**Figure 2:**
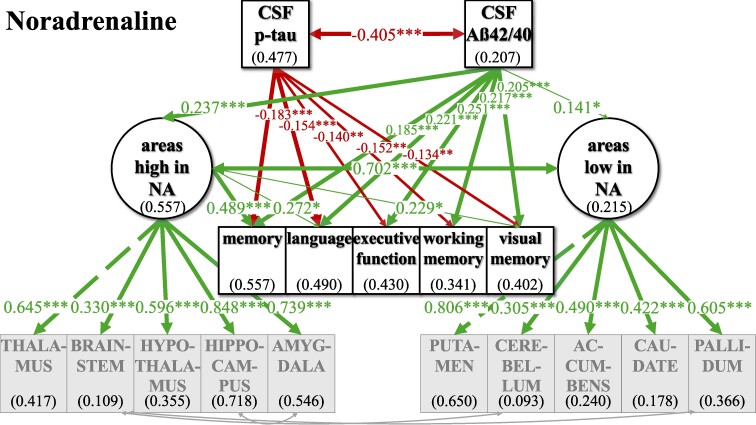
**SEM model for the whole group analyses (*n* = 400) for the noradrenergic system.** The respective included brain areas are shown estimating latent factors ‘areas high in NA and ‘areas low in NA’. Light grey arrows indicate added intercorrelations between brain regions based on the top three modification indices for model fit improvement. CSF biomarkers p-tau and Aß42/40 are inversely; ‘areas high in NA’ and ‘areas low in NA’ are positively correlated. p-tau is not significantly linked to ‘areas high in NA’ and ‘areas low in NA’, while Aß42/40 shows positive links to ‘areas high in NA’ and ‘areas low in NA’. ‘Areas high in NA’ were only significantly positively linked to the cognitive variables memory, language and visual memory. For an overview of regressions across neurotransmitters, see Tables 2 and 3. Not shown here for representational clarity: CSF biomarkers, latent factors and cognitive variables were controlled for additional AD risk factors (see Supplementary Table 3 for estimates of relationships). Latent factors are shown in ellipses, and observed variables are shown in squares. Numbers given in ellipses and squares indicate *R*^2^ of explained variance in the respective dependent variable. Only significant links are shown. ***P ≤ 0.001. **P ≤ 0.01. *P ≤ 0.05.

**Figure 3:**
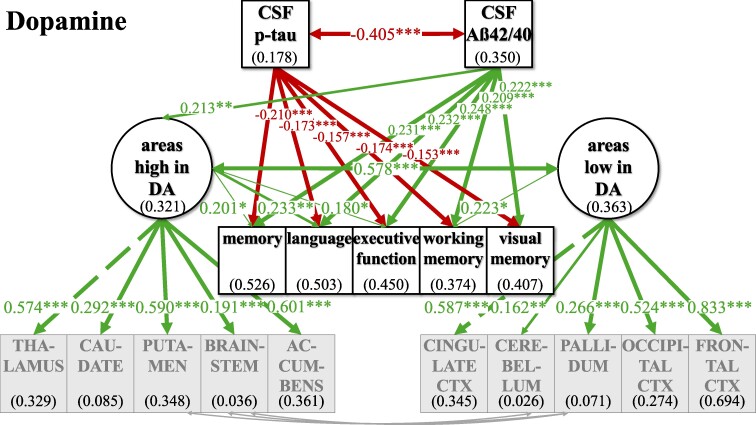
**SEM model for the whole group analyses (*n* = 400) for the dopaminergic system.** The respective included brain areas are shown estimating latent factors ‘areas high in DA’ and ‘areas low in DA’. Light grey arrows indicate added intercorrelations between brain regions based on the top three modification indices for model fit improvement. CSF biomarkers p-tau and Aß42/40 are inversely; ‘areas high in DA’ and ‘areas low in DA’ are positively correlated. p-tau is not significantly linked to ‘areas high in DA’ and ‘areas low in DA’, while Aß42/40 shows a positive link to ‘areas high in DA’, but not to ‘areas low in DA’. ‘Areas high in DA’ were positively linked to memory, language and executive function, while ‘areas low in DA’ were positively linked to working memory. For an overview of regressions across neurotransmitters, see Tables 2 and 3. Not shown here for representational clarity: CSF biomarkers, latent factors and cognitive variables were controlled for additional AD risk factors (see Supplementary Table 3 for estimates of relationships). Latent factors are shown in ellipses, and observed variables are shown in squares. Numbers given in ellipses and squares indicate *R*^2^ of explained variance in the respective dependent variable. Only significant links are shown. CTX, cortex. ***P ≤ 0.001. **P ≤ 0.01. *P ≤ 0.05.

**Figure 4:**
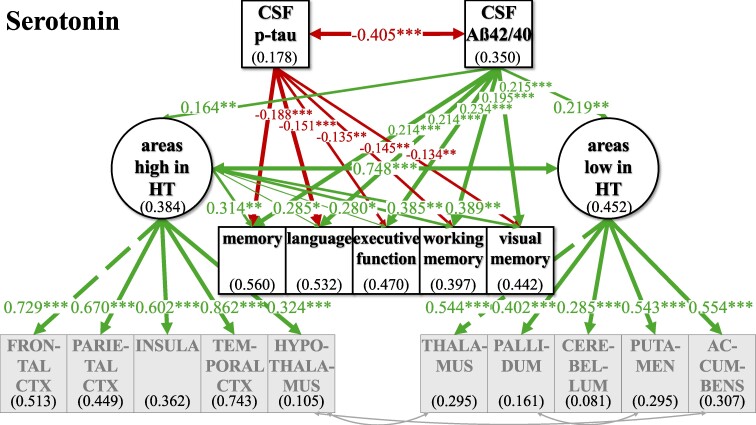
**SEM model for the whole group analyses (*n* = 400) for the serotonergic system.** The respective included brain areas are shown estimating latent factors ‘areas high in HT’ and ‘areas low in HT’. Light grey arrows indicate added intercorrelations between brain regions based on the top three modification indices for model fit improvement. CSF biomarkers p-tau and Aß42/40 are inversely; ‘areas high in HT’ and ‘areas low in HT’ are positively correlated. p-tau is not significantly linked to ‘areas high in HT’ and ‘areas low in HT’, while Aß42/40 shows positive links to ‘areas high in HT’ and ‘areas low in HT’. ‘Areas high in HT’ showed a positive link to all cognitive factors, while ‘areas low in HT’ were not significantly linked to any of the cognitive factors. For an overview of regressions across neurotransmitters, see Tables 2 and 3. Not shown here for representational clarity: CSF biomarkers, latent factors and cognitive variables were controlled for additional AD risk factors (see Supplementary Table 3 for estimates of relationships). Latent factors are shown in ellipses, and observed variables are shown in squares. Numbers given in ellipses and squares indicate *R*^2^ of explained variance in the respective dependent variable. Only significant links are shown. CTX, cortex. ***P ≤ 0.001. **P ≤ 0.01. *P ≤ 0.05.

**Figure 5:**
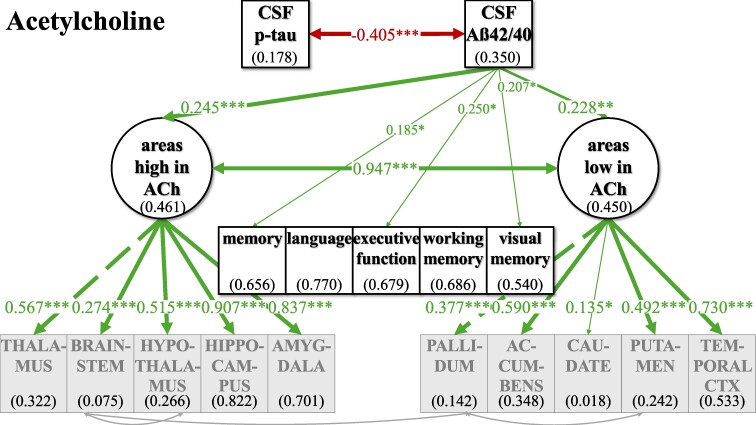
**SEM model for the whole group analyses (*n* = 400) for the cholinergic system.** The respective included brain areas are shown estimating latent factors ‘areas high in ACh’ and ‘areas low in ACh’. Light grey arrows indicate added intercorrelations between brain regions based on the top three modification indices for model fit improvement. CSF biomarkers p-tau and Aß42/40 are inversely; ‘areas high in ACh’ and ‘areas low in ACh’ are positively correlated. p-tau is not significantly linked to ‘areas high in ACh’ and ‘areas low in ACh’, while Aß42/40 shows positive links to ‘areas high in ACh’ and ‘areas low in ACh’. Neither ‘areas high in ACh’ nor ‘areas low in ACh’ were significantly linked to cognition. For an overview of regressions across neurotransmitters, see Tables 2 and 3. Not shown here for representational clarity: CSF biomarkers, latent factors and cognitive variables were controlled for additional AD risk factors (see Supplementary Table 3 for estimates of relationships). Latent factors are shown in ellipses, and observed variables are shown in squares. Numbers given in ellipses and squares indicate *R*^2^ of explained variance in the respective dependent variable. Only significant links are shown. CTX, cortex. ***P ≤ 0.001. **P ≤ 0.01. *P ≤ 0.05.

The standard pipeline of ‘FreeSurfer’ (version 7.1) was used for automated segmentation of subcortical brain structures and parcellation of cortical areas to obtain volume measures. Selection of brain areas was based on mean cut-off values for the normalized expression of transcripts per million (nTPM) for the respective receptors from the Human Protein Atlas.^37^ Sixteen cortical and subcortical brain areas (see Supplementary Table 1) were ranked according to the distribution values of the different receptors (e.g. for NA ADRA1A, ADRA1B, ADRA1D, ADRA2A, ADRA2B, ADRA2C, ADRB1, ADRB2, ADRB3), for each neurotransmitter. Different sensitivities of receptors to neurotransmitter levels within one neurotransmitter system was taken into account by ranking each receptor separately before brain areas high and low in receptors based on the five highest and five lowest sum scores of rankings across receptors. ‘Areas high in NA’ were identified as thalamus, brainstem, hypothalamus, hippocampus and amygdala, while ‘areas low in NA’ were identified as putamen, cerebellum, nucleus accumbens, caudate and pallidum (Fig. 2). ‘Areas high in DA’ were defined as thalamus, caudate, putamen, brainstem and nucleus accumbens; ‘areas low in DA’ were defined as cingulate cortex, cerebellum, pallidum, occipital cortex and frontal cortex (Fig. 3). ‘Areas high in HT’ were defined as frontal cortex, parietal cortex, insula, temporal cortex and hypothalamus; ‘areas low in HT’ were defined as thalamus, pallidum, cerebellum, putamen and nucleus accumbens (Fig. 4). ‘Areas high in ACh’ were defined as thalamus, brainstem, hypothalamus, hippocampus and amygdala; ‘areas low in ACh’ were defined as pallidum, nucleus accumbens, caudate, putamen and temporal cortex (Fig. 5). The separation into these extreme groups showed some expected overlaps for the different neurotransmitters that ranged from overlapping for all but one region (low neurotransmitter areas for NA and ACh (cerebellum/temporal cortex)] to no overlap at all (high neurotransmitter areas in DA and HT). Volumes were averaged across hemispheres using Sequence Adaptive Multimodal SEGmentation, implemented in the FreeSurfer 7.1 distribution. White matter hyperintensities (WMHs) were analysed in T_2_-weighted FLAIR images with the Lesion Segmentation Toolbox at a threshold of 0.5 and measured as total lesion volume in millilitres (*n* = 870). Obtained values for WMH, total grey matter volume (TGV) and regional volumes were corrected for TIV:


(1) $$\mathrm{WMH}(\mathrm{corrected})=\frac{\mathrm{total}\;\mathrm{white}\;\mathrm{matter}\;\mathrm{hyperintensity}\;\mathrm{volume}}{\mathrm{total}\;\mathrm{intracranial}\;\mathrm{volume}}$$

(2) $$\mathrm{TGV}(\mathrm{corrected})=\frac{\mathrm{total}\;\mathrm{grey}\;\mathrm{matter}\;\mathrm{volume}}{\mathrm{total}\;\mathrm{intracranial}\;\mathrm{volume}}.$$

### Statistical analyses

In order to allow fitting SEMs with similar range of variance to heterogeneous data inputs (e.g. demographic data, brain imaging data), variables were adjusted to values between 0 and 10.^38-40^ All brain volume variables were normally distributed. For *ApoE4* genotyping, alleles 2/2, 2/3 and 3/3 were defined as a negative carrier status whilst 2/4, 3/4 and 4/4 alleles were considered *ApoE4* carrier positive. Statistical analyses were performed in ‘R Statistical Software Package version 2022.12.0+353’^41^ using the ‘lavaan’ software package.^42^ For reliable results and parameter estimates in SEM depending on the sample size, a ratio of *N* to the number of parameters of 5:1 is recommended,^43^ which is sufficiently achieved with our sample size of 400 subjects and 10 observed variables estimating one or two latent factors.

The model fit was evaluated using incremental and absolute fit indices, considering the model’s complexity, sample size and degrees of freedom. The incremental fit indices, comparative fit index (CFI) and Tucker–Lewis index (TLI) are independent of sample size but suffer from lower average correlations between variables in heterogeneous data sets. Absolute fit indices, root mean square error of approximation (RMSEA) and standardized root mean square residual (SRMR), are worse (i.e. higher) in data sets with small sample sizes and low degrees of freedom. RMSEA describes the variance and covariance discrepancies from the model’s values.^44^ Model fits between non-nested models were assessed using ANOVA *χ*^2^ difference test and compared based on Akaike’s information criterion (AIC) and Bayesian information criterion (BIC). Intergroup differences were investigated using multiple group comparisons while aiming for measurement invariance of factors high and low in neurotransmitters across disease groups (see Supplementary Fig. 1 for details). Measurement invariance was tested by fixing factor loadings of contributing brain areas to the two factors to be the same across groups. Group differences in correlations between CSF biomarkers, cognitive factors and latent factors were tested using ANOVA likelihood ratio tests and assumed if models with specific regressions fixed to be equal across groups yielded worse fits.^45^

**Figure 6:**
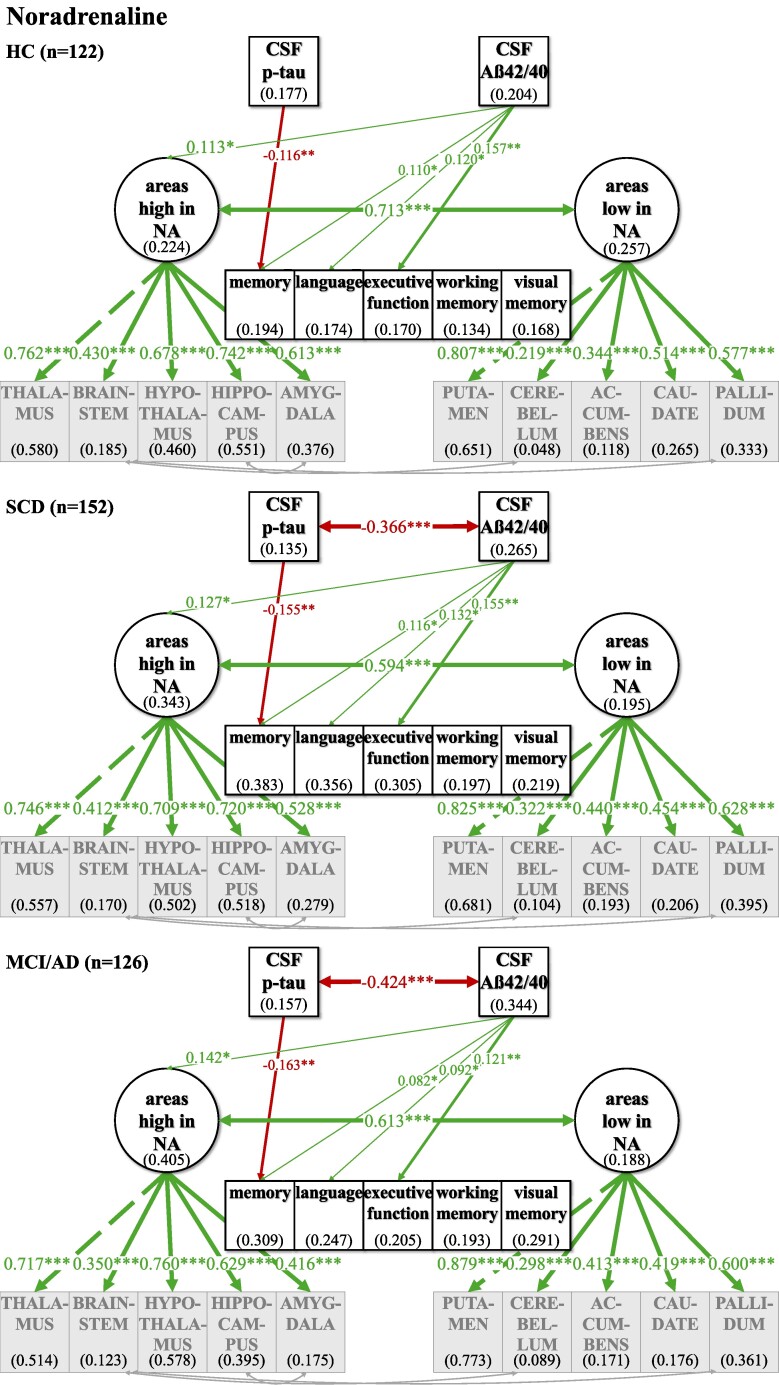
**Multiple group comparison SEM models for NA comparing HC and relatives, SCD and MCI and AD patients.** CSF biomarkers p-tau and Aß42/40 differed significantly in their correlation across disease severity groups. Phosphorylated tau levels and ratio of Aß42/40 were inversely associated in the SCD and MCI/AD group while no significant association could be observed in the healthy group. ‘Areas high in NA’ and ‘areas low in NA’ are strongly positively correlated across individuals in all subgroup analyses. Higher amyloid pathology indicated atrophy in ‘areas high in NA’. This link was not significantly differing across groups but showed a trend of stronger association with disease severity. Numbers given in ellipses and squares indicate *R*^2^ of explained variance in the respective dependent variable. Group differences were tested within the multiple group comparison using *χ*^2^ difference test with a *P* ≤ 0.5. ***P ≤ 0.001. **P ≤ 0.01. *P ≤ 0.05.

**Figure 7:**
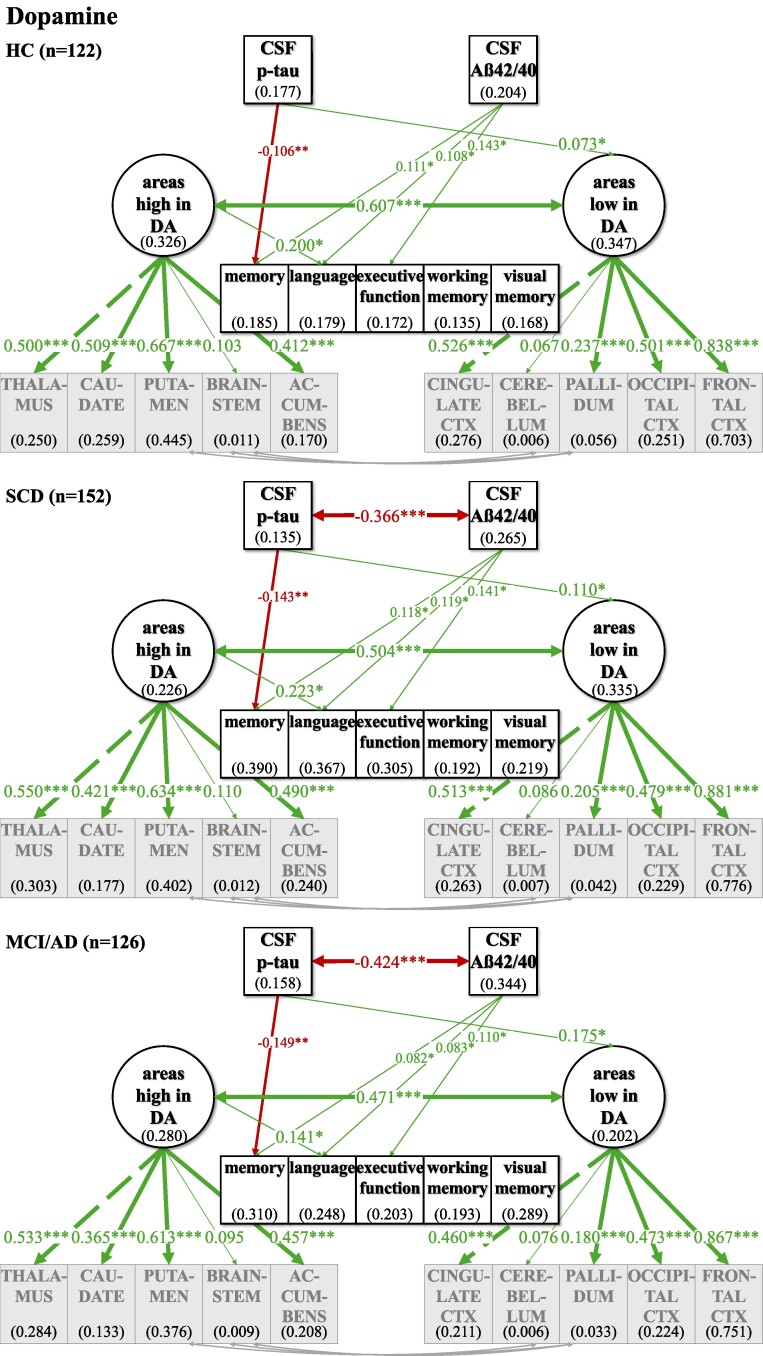
**Multiple group comparison SEM models for dopamine comparing HC and relatives, SCD and MCI and AD patients.** CSF biomarkers p-tau and Aß42/40 differed significantly in their correlation across disease severity groups. Phosphorylated tau levels and ratio of Aß42/40 were inversely associated in the SCD and MCI/AD group while no significant association could be observed in the healthy group. ‘Areas high in DA’ and ‘areas low in DA’ are strongly positively correlated across individuals in all subgroup analyses. p-tau shows a positive link to ‘areas low in DA’ without significant difference between disease severity groups but showing a trend with disease severity of a stronger association of higher tau pathology with higher volumes in ‘areas low in DA’. Numbers given in ellipses and squares indicate *R*^2^ of explained variance in the respective dependent variable. Group differences were tested within the multiple group comparison using *χ*^2^ difference test with a *P* ≤ 0.5. CTX, cortex. ***P ≤ 0.001. **P ≤ 0.01. *P ≤ 0.05.

**Figure 8:**
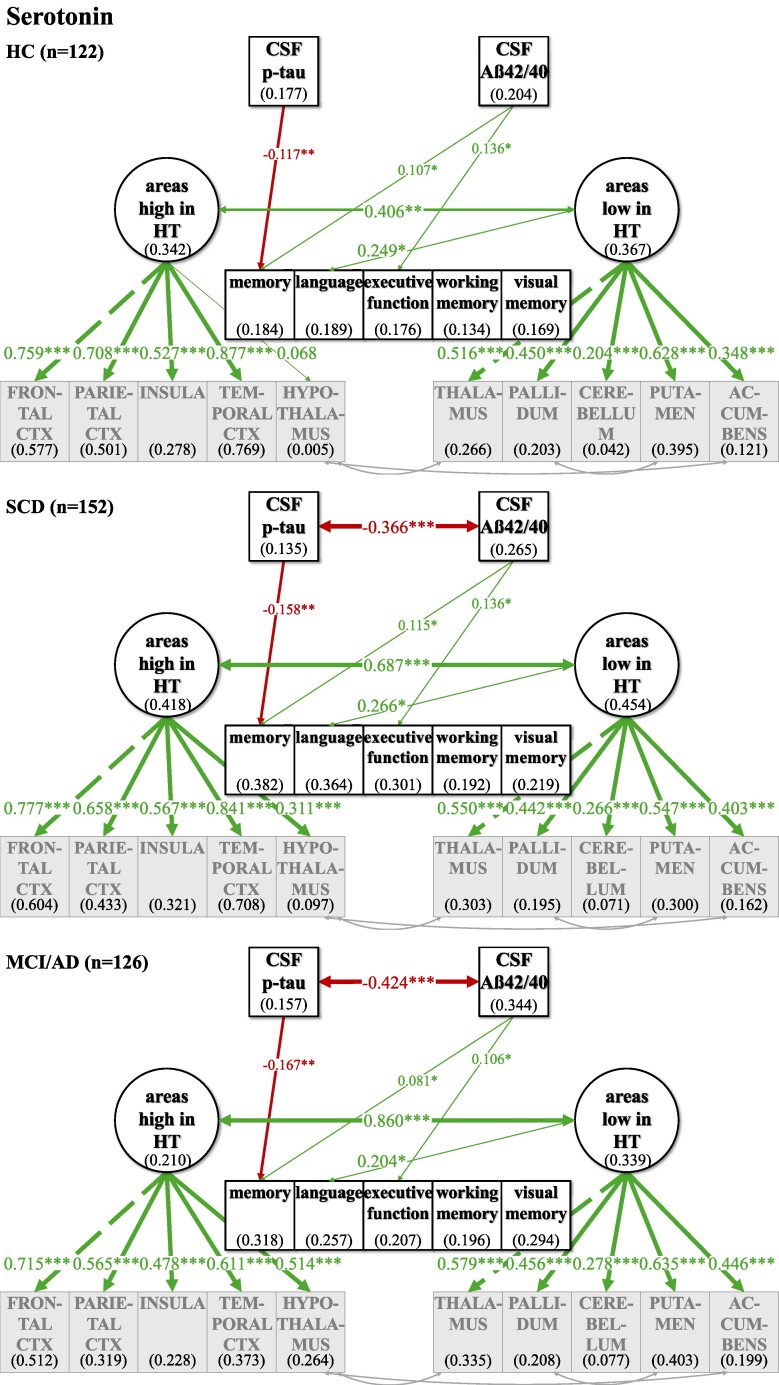
**Multiple group comparison SEM models for serotonin comparing HC and relatives, SCD and MCI and AD patients.** CSF biomarkers p-tau and Aß42/40 differed significantly in their correlation across disease severity groups. Phosphorylated tau levels and ratio of Aß42/40 were inversely associated in the SCD and MCI/AD group while no significant association could be observed in the healthy group. ‘Areas high in HT’ and ‘areas low in HT’ are strongly positively correlated across individuals in all subgroup analyses, while this association differed across groups, with a stronger association with disease severity. Numbers given in ellipses and squares indicate *R*^2^ of explained variance in the respective dependent variable. Group differences were tested within the multiple group comparison using *χ*^2^ difference test with a *P* ≤ 0.5. CTX, cortex. ***P ≤ 0.001. **P ≤ 0.01. *P ≤ 0.05.

A total of 3 SEMs were tested in order to assess differential relationships of ‘areas high and low’ in neurotransmitter systems: Firstly, we compared a unidimensional model summarizing all selected brain area volumes in one factor to a model separating ‘areas high’ and ‘areas low’ in receptor density for the respective neurotransmitter. Secondly, we assessed the relationship of brain volumes in ‘areas high and low’ to clinical and cognitive variables relevant to aging and Alzheimer’s disease (‘CSF cognitive model’, Figs 2–5) by adding CSF biomarkers (p-tau, ratio Aß42/40), clinical cognitive covariates (memory, language, executive functions, working memory and visual memory) and external variables controlling for AD risk (age, gender, education, *ApoE4* status, WMH). Finally, intergroup differences (HC, SCD and MCI/AD) in regressions between CSF biomarkers and latent factors ‘areas high and low’, latent factors ‘areas high and low’ and clinical cognitive covariates, CSF biomarkers and clinical cognitive covariates as well as correlations between CSF biomarkers and latent factors were investigated in the ‘CSF cognitive model’ using multiple group comparisons (Figs 6–8). In doing so, metric measurement invariance of the latent factors’ estimation for ‘areas high’ and ‘areas low’ was tested when factor loadings were fixed to be the same across all three groups (see Supplementary Fig. 1). If measurement invariance across the disease groups was not found, we checked with Lagrange multiplier tests that factor loadings needed to be freely estimated and set up a model with partial metric invariance as recommended.^43^

## Results

### Sample description

Demographic information on the three groups and between-group differences is found in Table 1. Groups differed significantly in age with older subjects in the SCD and MCI/AD group. As expected, in MCI/AD, we saw significantly more *ApoE4* carriers (49.2%) than in the SCD (32.2%) or healthy group (24.6%). Education was significantly lower in MCI/AD (mean ± SD = 4.3 ± 2.02) than in SCD (mean ± SD = 5.3 ± 1.89) or healthy subjects (mean ± SD = 5.11 ± 1.82). The amount of WMH was significantly higher in individuals diagnosed with MCI/AD compared to the SCD or healthy group. All groups differed significantly in TGV with lower volumes in the MCI/AD compared to the SCD group, and highest volumes in the healthy group. The MCI/AD group showed significantly higher values for p-tau and lower values for Aß42/40 compared to SCD and healthy subjects, while no difference in CSF biomarkers between the SCD and healthy group was observed. Cognitive function—reflected by memory, language, executive function, working memory and visual memory—was also lower in the MCI/AD group compared to the other groups. No group differences for gender or hypertension status were observed.

**Table 1:** Cohort description and group differences for variables age, gender, education, ApoE4 carrier status, WMH, TGV, amyloid-ß 42/40, p-tau and memory tested by ANOVA

Variable	HC (*n* = 122)	SCD (*n* = 152)	MCI/AD (*n* = 126)
Age (years, mean ± SD)^a^	67.75 ± 4.97	70.35 ± 5.77^b^	72.88 ± 5.67^b,c^
Gender (% female)	45.9	56.6	47.6
Education (mean ± SD)^a^	5.11 ± 1.82	5.30 ± 1.89	4.30 ± 2.02^b,c^
ApoE4 carrier status (% positive)^a^	24.6	32.2	49.2^b,c^
WMH (nL, mean ± SD)^a^	26.4 ± 70.8	36.3 ± 60.1	75.2 ± 101.4^b,c^
Hypertension (% present)	49.6	53.4	60.9
TGV (mean ± SD)^a^	597,674.1 ± 51 739.95	604 032.1 ± 51 670.1	563 413.0 ± 51 238.82^b,c^
Amyloid-ß 42/40 (mean ± SD)^a^	0.1 ± 0.02	0.09 ± 0.03	0.07 ± 0.03^b,c^
p-tau (mean ± SD)^a^	49.36 ± 17.2	52.64 ± 24.87	76.34 ± 40.57^b,c^
Cognition score (mean ± SD)^a^	0.53 ± 0.48	0.4 ± 0.51	−1.22 ± 0.83^b,c^

Groups were assigned to healthy subjects and first-degree relatives to AD (HC group), subjects with SCD (SCD group) and mild cognitive impaired and Alzheimer’s disease patients (MCI/AD group).

^a^Significantly different between groups.

^b^Significantly different compared to HC.

^c^Significantly different compared to SCD.

### Establishing difference between ‘areas high’ and ‘areas low’

For all neurotransmitters, the two-factor model with separate latent factors representing ‘areas high’ from ‘areas low’ revealed a better fit for our data compared to the unidimensional model that estimated one common latent factor comprising all observed brain area volumes (Supplementary Fig. 2). Better fit between the non-nested models was defined as lower AIC and BIC in the two-factor models, although the BIC was equal for the ACh one- and two-factor models (Supplementary Table 2). Regarding ACh, one- and two-factor models were comparatively more similarly likely given the data given BIC—which penalizes more complex models—did not show a potentially slightly better fit of the two-factor model. Model fit was evaluated, and the modification indices were checked for fit improvement based on heterogenous covariances between specific brain area volumes. In each two-factor model, three intercorrelations between specific brain area volumes were added (Figs 2–5; for details see Supplementary Fig. 2).

### Investigating relations of latent factors with CSF biomarkers and cognitive function in the whole sample

In our final ‘CSF cognition models’ (Figs 2–5), CSF biomarkers p-tau and Aß42/40 as well as cognitive variables were added while controlling for confounding variables of Alzheimer’s disease risk as outlined above. The model fit showed significant *χ*^2^ for test statistics (*P* < 0.001) and acceptable incremental and absolute parameters for the whole sample analysis in all neurotransmitter models (Table 2). As the incremental fit parameters CFI and TLI depend on the average correlation of the variables in the data set, a likely explanation for the somewhat lower CFI and TLI are the low and partially non-significant correlations between measures of brain volume after TIV correction (Supplementary Fig. 3). Nonetheless, TIV correction is necessary to control for interindividual differences in overall brain size while investigating the relevance of selective volume differences in ‘areas high and low’. Despite lower coherence amongst the brain volume measures, almost all factor loadings of the standardized estimates of the brain area volumes on latent factors ‘areas high and low’ were >0.3, indicating a reasonable amount of variance contribution to estimate the latent factors. Effect sizes for links of the control variables age, gender, *ApoE4* positivity, education and WMH on CSF biomarkers, cognitive variables and the latent factors ‘areas high’ and ‘areas low’ are listed in Supplementary Table 3.

**Table 2:** SEM model results for the whole sample models and the multiple group analysis for each neurotransmitter showing model fit parameters and effect sizes for associations between pathology markers, latent variables ‘areas high and low’ and latent variables and cognitive variables

	NA				DA				HT				ACh			
	Whole sample (*n* = 400)	HC + relatives (*n* = 122)	SCD (*n* = 152)	MCI/AD (*n* = 126)	Whole sample (*n* = 400)	HC + relatives (*n* = 122)	SCD (*n* = 152)	MCI/AD (*n* = 126)	Whole sample (*n* = 400)	HC + relatives (*n* = 122)	SCD (*n* = 152)	MCI/AD (*n* = 126)	Whole sample (*n* = 400)	HC + relatives (*n* = 122)	SCD (*n* = 152)	MCI/AD (*n* = 126)
CFI	0.918	0.904			0.922	0.892			0.940	0.916			0.921	NA		
TLI	0.857	0.858			0.864	0.840			0.896	0.875			0.863	NA		
RMSEA	0.095	0.080			0.087	0.080			0.079	0.073			0.094	NA		
SRMR	0.081	0.086			0.072	0.088			0.057	0.077			0.068	NA		
p-tau ↔ Aß42/40	−**0**.405***	−0.119ª	−**0**.366***^,^ª	−**0**.424***^,^ª	−**0**.405***	−0.119ª	−**0**.366***^,^ª	−**0**.424***^,^ª	−**0**.405***	−0.119ª	−**0**.366***^,^ª	−**0**.424***^,^ª	−**0**.405***	NA	NA	NA
Areas high ↔ areas low	**0**.702***	**0**.713***	**0**.594***	**0**.613***	**0**.578***	**0**.607***	**0**.504***	**0**.471***	**0**.784***	**0**.406**^,^ª	**0**.687***^,^ª	**0**.860***^,^ª	**0**.947***	NA	NA	NA
p-tau → areas high	−0.066	0.004	0.006	0.010	−0.059	−0.027	−0.034	−0.056	−0.052	0.035	0.053	0.082	−0.082	NA	NA	NA
p-tau → areas low	−0.050	−0.010	−0.011	−0.018	0.073	**0**.073*	**0**.110*	**0**.175*	−0.062	0.006	0.007	0.010	−0.108	NA	NA	NA
Aß42/40 → areas high	**0**.237***	**0**.113*	**0**.127*	**0**.142*	**0**.213**	0.117	0.115	0.127	**0**.164**	0.037	0.044	0.046	**0**.245***	NA	NA	NA
Aß42/40 → areas low	**0**.141*	0.071	0.066	0.069	0.103	0.015	0.018	0.019	**0**.219**	0.114	0.119	0.108	**0**.228**	NA	NA	NA
Areas high → memory	**0**.489***	0.156	0.147	0.092	**0**.201*	0.171	0.185	0.118	**0**.314**	−0.010	−0.009	−0.006	−0.691	NA	NA	NA
Areas high → language	**0**.272*	−0.004	−0.004	−0.003	**0**.233**	**0**.200*	**0**.223*	**0**.141*	**0**.285*	−0.066	−0.062	−0.042	−1.730	NA	NA	NA
Areas high → executive function	0.150	−0.065	−0.057	−0.040	**0**.180*	0.160	0.161	0.114	**0**.280*	−0.050	−0.042	−0.031	−1.726	NA	NA	NA
Areas high → working memory	0.086	−0.127	−0.114	−0.083	0.101	0.024	0.024	0.018	**0**.358**	0.096	0.081	0.064	−2.127	NA	NA	NA
Areas high → visual memory	**0**.229*	−0.044	−0.041	−0.030	0.079	−0.020	−0.022	−0.016	**0**.389**	0.072	0.064	0.049	−1.211	NA	NA	NA
Areas low → memory	−0.081	0.017	0.020	0.013	0.148	−0.074	−0.066	−0.043	0.110	0.161	0.165	0.128	1.198	NA	NA	NA
Areas low → language	0.078	0.143	0.169	0.113	0.146	−0.060	−0.055	−0.036	0.164	**0**.249*	**0**.266*	**0**.204*	2.185	NA	NA	NA
Areas low → executive function	0.122	0.159	0.169	0.126	0.159	−0.033	−0.027	−0.020	0.105	0.192	0.183	0.157	2.089	NA	NA	NA
Areas low → working memory	0.110	0.114	0.124	0.097	**0**.223*	0.087	0.073	0.056	−0.002	0.022	0.021	0.019	2.446	NA	NA	NA
Areas low → visual memory	−0.034	0.020	0.023	0.017	0.147	−0.011	−0.010	−0.008	−0.114	−0.039	−0.039	−0.035	1.509	NA	NA	NA

Bold values show significant associations. NA, not available.

***P ≤ 0.001, **P ≤ 0.01, *P ≤ 0.05.

ªGroups differ.

For an overview of effects in the respective CSF cognition models, see Figs 2–5 and Tables 2 and3. As expected, CSF biomarkers were inversely correlated (*ß* = −0.405; *P* ≤ 0.001), indicating that individuals with more tau pathology also showed more amyloid pathology. Similarly, ‘areas high’ and ‘areas low’ were positively correlated for each neurotransmitter (NA: *ß* = 0.702; *P* ≤ 0.001; DA: *ß* = 0.578; *P* ≤ 0.001; HT: *ß* = 0.748; *P* ≤ 0.001; ACh: *ß* = 0.947; *P* ≤ 0.001), indicating that individuals with less (TIV-corrected) atrophy in ‘areas high’ in receptor densities also had less atrophy in ‘areas low’.

**Table 3:** SEM model results for the whole sample and the multiple group analysis for each neurotransmitter for the link between biomarkers and cognitive variables

	NA				DA				HT				ACh			
	Whole sample (*n* = 400)	HC + relatives (*n* = 122)	SCD (*n* = 152)	MCI/AD (*n* = 126)	Whole sample (*n* = 400)	HC + relatives (*n* = 122)	SCD (*n* = 152)	MCI/AD (*n* = 126)	Whole sample (*n* = 400)	HC + relatives (*n* = 122)	SCD (*n* = 152)	MCI/AD (*n* = 126)	Whole sample (*n* = 400)	HC + relatives (*n* = 122)	SCD (*n* = 152)	MCI/AD (*n* = 126)
p-tau → memory	−**0**.183***	−**0**.116**	−**0**.155**	−**0**.163**	−**0**.210***	−**0**.106**	−**0**.143**	−**0**.149**	−**0**.188***	−**0**.117**	−**0**.158**	−**0**.167**	−0.138	NA	NA	NA
p-tau → language	−**0**.154***	−0.070	−0.097	−0.102	−**0**.173***	−0.061	−0.085	−0.089	−**0**.151***	−0.070	−0.099	−0.104	−0.081	NA	NA	NA
p-tau → executive function	−**0**.140**	−0.062	−0.077	−0.090	−**0**.157***	−0.056	−0.070	−0.082	−**0**.135**	−0.062	−0.078	−0.092	−0.072	NA	NA	NA
p-tau → working memory	−**0**.152**	−0.061	−0.077	−0.095	−**0**.174***	−0.068	−0.086	−0.106	−**0**.145**	−0.066	−0.084	−0.103	−0.073	NA	NA	NA
p-tau → visual memory	−**0**.134**	−0.058	−0.077	−0.092	−**0**.153***	−0.057	−0.077	−0.091	−**0**.134**	−0.061	−0.081	−0.098	−0.083	NA	NA	NA
Aß42/40 → memory	**0**.185***	**0**.110*	**0**.116*	**0**.082*	**0**.231***	**0**.111*	**0**.118*	**0**.082*	**0**.214***	**0**.107*	**0**.115*	**0**.081*	**0**.185*	NA	NA	NA
Aß42/40 → language	**0**.221***	**0**.120*	**0**.132*	**0**.092*	**0**.232***	**0**.108*	**0**.119*	**0**.083*	**0**.214***	0.099	0.110	0.077	0.223	NA	NA	NA
Aß42/40 → executive function	**0**.251***	**0**.157**	**0**.155**	**0**.121**	**0**.248***	**0**.143*	**0**.141*	**0**.110*	**0**.234***	**0**.136*	**0**.136*	**0**.106*	**0**.250*	NA	NA	NA
Aß42/40 → working memory	**0**.217***	0.094	0.095	0.078	**0**.209***	0.084	0.085	0.070	**0**.195***	0.081	0.082	0.067	0.217	NA	NA	NA
Aß42/40 → visual memory	**0**.205***	0.100	0.106	0.085	**0**.222***	0.097	0.103	0.082	**0**.215***	0.096	0.103	0.082	**0**.207*	NA	NA	NA

Bold values show significant associations. NA, not available.

***P ≤ 0.001, **P ≤ 0.01, *P ≤ 0.05.

^a^Groups differ.

There was no significant link of p-tau on ‘areas high’ nor on ‘areas low’ in any of the neurotransmitter models. In contrast, Aß42/40 was linked, ‘areas high in NA’ (*ß* = 0.237; *P* ≤ 0.001) and ‘areas low in NA’ (*ß* = 0.141; *P* ≤ 0.05). Concerning the pathology biomarkers and their association to brain area volumes in the DA model, Aß42/40 only showed a positive link on ‘areas high in DA’ (*ß* = 0.213; *P* ≤ 0.01), but no significant association with ‘areas low in DA’. However, for HT and ACh, Aß42/40 was positively linked to ‘areas high’ (HT: *ß* = 0.164; *P* ≤ 0.01; ACh: *ß* = 0.245; *P* ≤ 0.001) and ‘areas low’ (HT: *ß* = 0.219; *P* ≤ 0.01; ACh: *ß* = 0.228; *P* ≤ 0.01).

Interesting differential links were observed with regard to links between cognition and volumes in ‘areas high’ and ‘areas low’ across the different neurotransmitters: ‘areas high in NA’ showed a positive link to memory (*ß* = 0.489; *P* ≤ 0.001), language (*ß* = 0.272; *P* ≤ 0.05) and visual memory (*ß* = 0.229; *P* ≤ 0.05), while ‘areas low in NA’ were not significantly linked to any of the cognitive variables. ‘Areas high in DA’ showed a positive link to memory (*ß* = 0.201; *P* ≤ 0.05), language (*ß* = 0.233; *P* ≤ 0.01) and executive function (*ß* = 0.180; *P* ≤ 0.05), while ‘areas low in DA’ were positively associated to working memory (*ß* = 0.223; *P* ≤ 0.05). ‘Areas high in HT’ were positively linked to all cognitive variables (memory: *ß* = 0.314; *P* ≤ 0.01; language: *ß* = 0.285; *P* ≤ 0.05; executive function: *ß* = 0.280; *P* ≤ 0.05; working memory: *ß* = 0.385; *P* ≤ 0.01; visual memory: *ß* = 0.389; *P* ≤ 0.01), while ‘areas low in HT’ did not show any significant link to cognitive functions. Also, neither ‘areas high in ACh’ nor ‘areas low in ACh’ were significantly linked to cognition.

For the neurotransmitter models of NA, DA and HT, the whole sample analysis showed similar links of CSF pathology markers p-tau and Aß42/40 on cognitive variables, indicating a clear decline in a wide range of cognitive functions. All cognitive variables were negatively linked to p-tau, suggesting a decrease of cognition by tau pathology, while additionally all cognitive variables showed a positive association to Aß42/40, indicating higher amyloid pathology with worse cognition across HCs, SCD and MCI/AD. However, in the ACh model, there were no such significant associations between p-tau and cognitive variables, but only between Aß42/40 and selective cognitive variables (memory, executive function, visual memory).

### Groups differ across Alzheimer’s disease spectrum in links of neurotransmitter target areas, biomarkers and cognition

In a second step, we used multigroup analyses within the ‘CSF cognition model’ to examine group differences in the above observed relationships, as correlations in the whole group between e.g. cognitive function and pathology will not differentiate links based on mean differences ‘between’ groups and interindividual differences ‘within’ groups. In order to allow for sufficient sample sizes within each group, three groups were formed: HCs and healthy first-degree relatives to Alzheimer’s patients (*n* = 122), individuals with SCD (*n* = 152) and MCI and mild Alzheimer’s disease dementia (*n* = 126). This multiple group comparison did not converge for the ACh model likely due to the high correlation between ‘areas high’ and ‘areas low’ (*ß* = 0.947; *P* ≤ 0.001), that is, a less well fitting two-factor model (see also section “Establishing difference between ‘areas high’ and ‘areas low’” and Supplementary Table 2). When assessing degrees of measurement invariance across groups with regard to the latent factors ‘areas high and low’, for the HT model, groups showed partial metric measurement invariance for the latent factors only when the factor loading of ‘areas high in HT’ on hypothalamus was freely estimated, while in the NA and DA models, all factor loadings of specific anatomical areas could be fixed to be equal across groups to achieve weak measurement invariance. For NA, in all groups, all observed variables loaded significantly and positively on the respective latent factors, while for DA, brainstem and cerebellum did not load significantly on ‘areas high and low’ in any group and for HT hypothalamus did not load significantly on ‘areas high’ only for the healthy subjects. Taken together, ‘areas high and low’ thus represent the same factors across groups except for a lower contribution of hypothalamic volumes to the definition of ‘areas high in HT’ in the healthy group. For DA, the multiple group comparison showed that within each group, interindividual differences in brainstem and cerebellum volume do not significantly define ‘areas high and low’, suggesting a reduced contribution of brainstem and cerebellum volumes to explaining interindividual differences as compared to mean group differences.

Effect sizes for all correlation and regression paths of the structural model and for each neurotransmitter and the respective group are shown in Tables 2 and 3 and Supplementary Table 2. As can be seen in Figs 6–8 and Table 2, the correlation across individuals between the CSF pathology biomarkers p-tau and Aß 42/40 differed across groups, with no significant association in the healthy group, while the SCD and MCI/AD groups showed a positive association between higher tau and amyloid pathology. Links between pathology markers thus also hold information for differentiating more affected groups based on patterns in interindividual differences. While mean levels of pathologies were as expected significantly higher in MCI/AD (see Table 1), this suggests that differences between individuals that are more and less affected by pathologies in those two groups might be comparable. Moreover, HC and SCD did not differ in mean levels of pathologies, but only in the SCD group individuals differed significantly in their relationship. As in the whole sample analysis, volumes in ‘areas high and low’ were positively correlated across individuals within the disease groups. Solely for HT, groups differed significantly in their correlation showing a stronger association of ‘areas high and low’ with disease severity across individuals in healthy subjects (*ß* = 0.406; *P* ≤ 0.01), SCD (*ß* = 0.687; *P* ≤ 0.001) and MCI/AD patients (*ß* = 0.860; *P* ≤ 0.001).

Concerning the associations between CSF biomarkers with ‘areas high and low’, p-tau was not linked to any of the latent factors across individuals within any of the groups for any neurotransmitter, similar to the analysis in the whole sample. Except for dopamine, where p-tau was significantly associated with ‘areas low in DA’, while not differing significantly across groups but showing a trend of stronger association across individuals within groups with increasing disease severity. Deviating from the whole sample analysis, the group comparison revealed no association across individuals within groups of Aß42/40 with ‘areas high and low’, except for the NA system, where individuals with higher amyloid pathology showed atrophy in ‘areas high in NA’, also within disease severity groups. However, the positive association between Aß42/40 and ‘areas low in NA’, observed in the whole sample analysis, was not evident in interindividual differences in the NA subgroup analysis.

The negative associations in the whole group analysis of p-tau with each cognitive variable (Figs 2–4) only persisted significantly for memory function (Figs 6–8), suggesting that only with regard to memory, increased tau pathology can explain interindividual differences in lower memory within groups. Similarly, interindividual differences in amyloid burden were able to explain interindividual differences in all three groups only with regard to memory, language and executive function for NA, DA and HT (for the latter neurotransmitter, the link to language did not reach significance within groups).

Finally, in the NA subgroup analysis across individuals within groups, no significant link of ‘areas high or low in NA’ to cognitive performance could be observed, while the whole group analysis showed positive associations of ‘areas high in NA’ with memory, language and visual memory. In the DA model, ‘areas high in DA’ were positively linked only to language across individuals in all subgroups, compared to significant positive associations of ‘areas high in DA’ with memory, language and executive function and ‘areas low in DA’ with working memory in the whole sample analysis. Furthermore, ‘areas low in HT’ showed a positive association across individuals with language in all subgroups, which was not observed in the whole sample analysis.

Taken together, tau pathology predicted memory performance and amyloid pathology predicted memory performance and executive function in all subgroups. In the NA model, amyloid pathology was especially associated with ‘areas high in NA’, while in the DA model, tau pathology was especially associated with ‘areas low in DA’.

## Discussion

Our study aimed to better understand how structural alterations in neurotransmitter systems are related to healthy aging and disease severity in Alzheimer’s disease. As the brain structures supporting neurotransmitter systems appear to be particularly vulnerable to neurodegeneration, it may provide important information for differentiating cognitive decline in healthy aging, SCD and Alzheimer’s disease dementia. Specifically, we investigated whether areas high or low in NA, DA, HT and ACh are differentially linked to CSF pathology markers (Aß42/40 and p-tau) and cognitive function. Relevance of a neurotransmitter for a specific area was defined based on differences in receptor densities across areas, with areas higher in receptor densities for a particular neurotransmitter considered ‘high’ and areas low in receptor densities considered ‘low’. We hypothesized that more intact areas that are high in neurotransmitter receptors may be indicative of a slower progression of the disease, and therefore, we expected to observe a stronger association between atrophy in these ‘areas high’ and higher Alzheimer’s disease pathology as well as lower cognitive function.

The observed and expected group differences in age, education, WMH and *ApoE4* positivity within an Alzheimer’s disease continuum have been described elsewhere. We controlled for them as external confounding variables when assessing the links between cognition, pathology markers and atrophy in high and low neurotransmitter brain areas as they likely relate to cognition, pathology levels as well as atrophy.

SEM confirmed our hypothesis that atrophies in brain areas separated into ‘areas high and low’ in NA, DA and HT neurotransmitter system progress differently across disease stages. For ACh, this separation was less clear, resulting also in difficulties in assessing interindividual differences and group differences in links of atrophies and cognition or pathology levels. The separation in the NA, DA and HT system suggests that there are separable disease-related atrophy trajectories of ‘areas high and low’. For NA, this could be due to a higher vulnerability of these areas with a decline in neurotransmitter support of vascular health and inflammation levels^14,15^ given that the NA system is affected by protein pathologies early on in Alzheimer’s disease and might show altered levels of neuromodulation as a consequence. Comprising the subregions of caudate, putamen and nucleus accumbens, the striatum is fully represented as an ‘area high in DA’ with high factor loadings around 0.6, except for caudate. Early degeneration with neuronal loss in the ventral tegmental area as the origin site for dopaminergic neurons has been described in Alzheimer’s disease resulting in reduced dopamine levels in its target areas such as hippocampus and nucleus accumbens, leading to memory decline.^46^ Similarly, Azargoonjahromi^47^ could recently show that higher serotonin levels are related to neurotrophic factors supporting neurogenesis and neuroplasticity as well as that serotonin is linked to higher cognitive function and larger brain volume. Post-mortem studies on Alzheimer’s disease patients showed reduced HT receptor binding in cortical areas—mainly the frontal cortex—as well as in the hippocampus and amygdala,^48^ suggesting cognitive decline and atrophy in line with our findings on ‘areas high in HT’.

As expected, individuals with more tau pathology also had higher levels of amyloid pathology in the whole sample, but with significantly stronger correlations with increasing disease severity. Given that no difference between the mean levels of tau and amyloid pathology for HC and SCD has been observed, it is interesting that the interrelatedness of pathology was higher in SCD as compared to HC and can therefore provide important additional information for assessing risk populations.

Atrophies in ‘areas high and low’ were positively correlated in all neurotransmitter models, indicating that interindividual differences in atrophies (even when controlling for TIV) are a further important marker for differentiating individuals. This correlation was particularly high in the cholinergic system, suggesting that a differentiation in ‘areas high and low’ in receptor levels is less appropriate for understanding the impact of a decline in the ACh system. Moreover, links between atrophies in ‘areas high and low’ did not differ across groups for the NA and DA system, but increased significantly across groups with disease severity for HT. This suggests a more differentiated decline of ‘areas high and low’ based on NA and DA receptor densities, while for HT, ‘areas high and low’ seem to exhibit a more synchronized decline.

We further hypothesized differential links between CSF biomarkers and atrophy in ‘areas high and low’, which we found for amyloid pathology, but not for tau pathology, except for subgroups in the dopaminergic model. In general, higher amyloid pathology was associated with atrophy in ‘areas high and low’ for the whole sample in all neurotransmitter models, except for dopamine, where this was only observed for areas high in receptor densities, but not for those low in densities. Within the whole sample, individuals with higher amyloid pathology showed more atrophy in ‘areas high in NA’ as well as in ‘areas low in NA’, whereas within disease severity groups, higher amyloid burden was only linked to ‘areas high in NA’. This might indicate an interaction between NA decline and amyloid burden in disease mechanisms, in line with post-mortem evidence that show amyloid plaques appear preferentially in NA high brain regions. However, whether this reflects causal links between NA dependency and amyloid pathologies remain to be investigated due to limited data on the longitudinal consequences of a structural decline in the NA system. It is unclear, for instance, whether early tau pathology in the noradrenergic LC results in reduced or temporarily increased NA release. The observed relationship of individuals with higher levels of amyloid pathology having lower brain volumes particularly in more NA-dependent areas could result from a reduced NA release due to higher vulnerability of NA target areas to Alzheimer’s disease pathology driven by their anti-inflammatory^14^ and blood flow regulating properties.^15^ On the other hand, it is also possible that NA release is (at least initially) not strongly reduced, and/or that brain areas more dependent on NA show relatively more resilience to protein pathologies which could also result in a stronger association of volume in NA-high areas and Alzheimer’s disease pathology across individuals. In summary, while we were able to identify a stronger association between NA-high versus NA-low areas with Alzheimer’s disease amyloid pathology, the underlying mechanism remains to be elucidated in the absence of longitudinal data including measures of functional NA release. Moreover, the link of higher tau pathology to less atrophy in ‘areas low in DA’ could point to a compensatory mechanism across Alzheimer’s disease progression in these regions but would have to be investigated longitudinally as well. Across disease groups, atrophy in ‘areas high in DA’ was higher with more amyloid burden, while ‘areas high and low’ in NA, HT and ACh did not show this differential relationship between ‘areas high and low’, suggesting a greater relevance of a decline in DA modulation. For instance, amyloid burden might be related to changes in perfusion in these amyloid loaded regions and dopamine depletion.^49^

Also with regard to links with cognitive data, differential links across neurotransmitters of specific cognitive functions with atrophies and pathology burden can be observed across disease progression, but also within groups. Investigating these links can inform on the relevance of cognitive markers for capturing specific aspects of disease progression as well as interindividual differences therein. Atrophy differences between groups were more frequently linked to differences in cognitive function in areas high in receptor densities, suggesting more cognition-related decline in areas more dependent on neurotransmitters investigated here. This was in particular the case for the cognitive functions memory, language, executive functions and visual memory. In terms of differentiating interindividual differences in atrophy also within groups, differences in language capacities were able to explain variance both in ‘areas high in DA’ and ‘areas low in HT’ in all subgroups. While language is not one of the main candidates for assessing disease severity in AD in cognitive assessments, this suggests that interindividual differences in neuromodulatory systems known to be affected in AD^9^ might impact language abilities.

As expected, higher levels of tau or amyloid burden markers showed a consistent negative relationship with all investigated cognitive functions across groups. This was not the case for ACh, likely due to a comparatively less suitable model setup as explained above. Examining interactions of pathology and cognition within groups, differential links were observed with p-tau, which explained interindividual differences in memory capacity in all three groups, and amyloid, which explained differences in memory, language and executive functions. This less specific link of amyloid burden with cognitive functions as compared to p-tau is in line with a more diffuse spread of amyloid pathologies.^50^ Our SEM approach allows investigating relative contributions of atrophies and pathology burden to cognitive function while controlling for known risk factors in aging and AD at the same time. Using this approach, we were able to show that pathology burden was more frequently linked to cognitive decline than atrophy (cf. Tables 2 and 3). This suggests that for a better understanding of the cognitive decline in neurodegeneration, measures of volumetric brain changes should be complemented by measures which are more sensitive to local levels of pathology burden such as PET or functional MRI assessments.

Taking together, from these results, we infer a greater relevance of atrophy in ‘areas high in NA and DA’ and changes in language function driven by amyloid pathology across the stages of Alzheimer’s disease, which should be further investigated in future clinical studies.

As a caveat to our results, although our sample size was sufficient for a SEM estimating two factors and controlling for external variables, it was necessary to combine clinical groups of the DELCODE study to achieve sufficient group sizes for the multiple group comparisons. We clustered the initially assigned five groups into three groups. In future studies, it would be interesting to distinguish the five different groups, especially disentangling the clinical group of MCI and Alzheimer’s disease patients. Moreover, our data did not fit the hypothesized model perfectly, but rather with borderline acceptable fit indices, likely due to low correlations in the raw data. Finally, to better understand the mechanisms underlying our correlational results, additional analyses in larger samples as well as stratification based on longitudinal markers will be necessary.

Overall, we hope that our study helps to highlight the importance of the neurotransmitter systems in healthy aging and the progression of Alzheimer’s disease. By employing a model estimation with SEM, we integrated non-invasive *in vivo* MRI measurements of brain areas that are more or less involved in specific neurotransmitter systems in humans. Our results suggest that the NA and DA system may be a key factor in resilience to onset of Alzheimer’s disease. Future studies should build on these findings and longitudinally explore how volumetric degeneration of individual neurotransmitter-specific brain areas predict cognitive decline across time.^51-54^

## Supplementary Material

fcaf031_Supplementary_Data

## Data Availability

DELCODE data are not publicly available, but may be provided from the corresponding authors, upon reasonable request. The code for the SEM in ‘lavaan’ is available on GitHub (https://github.com/lehaag/NA-SEM-model/blob/main/neurotransmitter-SEM-models).
