# Supplementary material for: CSF biomarkers are differentially linked to brain areas high and low in noradrenaline, dopamine and serotonin across the Alzheimer’s disease spectrum
Source: Brain Commun. 2025 Jan 23;7(1):fcaf031. doi: 10.1093/braincomms/fcaf031 (PMC11806415; doi:10.1093/braincomms/fcaf031)
Supplement: fcaf031_Supplementary_Data [file fcaf031_supplementary_data.docx]

| CEREBRAL CORTEX | **CINGULATE** | Anterior cingulate cortex, supragenual-dorsal |
| --- | --- | --- |
|  |  | Anterior cingulate cortex, supragenual-ventral |
|  |  | Middle cingulate cortex |
|  |  | Posterior cingulate cortex |
|  |  | Posterior cingulate cortex, dorsal |
|  |  | Posterior cingulate cortex, ventral |
|  | CLAUSTRUM | Claustrum |
|  | **FRONTAL** | Frontal eye field |
|  |  | Frontal operculum |
|  |  | Paracentral lobule, anterior |
|  |  | Precentral gyrus |
|  |  | Precentral gyrus, dorsal |
|  |  | Precentral gyrus, middle |
|  |  | Precentral gyrus, ventral |
|  |  | Premotor cortex, dorsal |
|  |  | Premotor cortex, ventral |
|  |  | Supplementary motor cortex |
|  |  | Orbitofrontal gyrus, anterior |
|  |  | Inferior frontal gyrus, opercular |
|  | **INSULAR** | Anterior insular cortex, dorsal |
|  |  | Anterior insular cortex, ventral |
|  |  | Posterior insular cortex |
|  | **OCCIPITAL** | Area parastriata, inferior |
|  |  | Area parastriata, parietal |
|  |  | Area parastriata, superior |
|  |  | Area parastriata, temporal |
|  |  | Area striata |
|  |  | Lingual gyrus |
|  |  | Occipital cortex |
|  |  | Parieto-occipital transitional area |
|  |  | Temporo-occipital transitional zone |
|  | PALEOCORTEX | Olfactory area |
|  |  | Olfactory tubercle |
|  |  | Piriform cortex |
|  | **PARIETAL** | Angular gyrus |
|  |  | Inferior parietal lobule |
|  |  | Intraparietal deep sulcus |
|  |  | Paracentral lobule, posterior |
|  |  | Parietal operculum |
|  |  | Parieto-insular cortex |
|  |  | Postcentral gyrus |
|  |  | Postcentral gyrus, dorsal |
|  |  | Postcentral gyrus, middle |
|  |  | Postcentral gyrus, ventral |
|  |  | Precuneus |
|  |  | Subcentral gyrus, S2 |
|  |  | Superior parietal lobule |
|  |  | Supramarginal gyrus |
|  | RETROSPLENIAL | Retrosplenial cortex |
|  | **TEMPORAL** | Fusiform gyrus |
|  |  | Inferior temporal gyrus |
|  |  | Middle temporal gyrus |
|  |  | Superior temporal gyrus |
|  |  | Temporal pole |
|  |  | Temporo-insular cortex, parainsular gyrus |
|  |  | Transversal temporal gyrus, anterior |
|  |  | Transversal temporal gyrus, posterior |
| **HIPPOCAMPUS** |  |  |
| **AMYGDALA** |  |  |
| BASAL GANGLIA | BASAL FOREBRAIN | Nucleus basalis of Meynert |
|  |  | Nucleus of the diagonal band |
|  |  | Septal nuclei |
|  |  | Stria terminalis, bed nucleus |
|  | **GLOBUS PALLIDUS** | Globus pallidus, externus |
|  |  | Globus pallidus, internus |
|  | STRIATUM | **Caudate nucleus** |
|  |  | **Nucleus accumbens** |
|  |  | **Putamen** |
| **THALAMUS** |  |  |
| **HYPOTHALAMUS** |  |  |
| **CEREBELLUM** |  |  |
| **BRAINSTEM** | MIDBRAIN |  |
|  | PONS |  |
|  | MEDULLA OBLONGATA | |

**Supplementary table 1: Selection of the brain areas**
Brain areas were selected from the Human Protein Atlas (<https://www.proteinatlas.org>, 18.11.2024) which reports data for the brain expression of the several receptors in ten superordinated cortical and subcortical gray matter regions (cerebral cortex, hippocampal formation, amygdala, basal ganglia, thalamus, hypothalamus, midbrain, cerebellum, pons and medulla oblongata). Information on the spinal cord, white matter and choroid plexus were not taken into consideration in this analysis. The table shows additionally the subordinated regions included in the particular brain areas. After checking for the representative regions extracted from the Freesurfer volume measures, the 16 **bolt** cortical and subcortical regions were included in the SEM models. The faint marked brain areas were not included in the analyses as separate volume data was not available from Freesurfer.

|  | | **NA** | **DA** | **HT** | **ACh** |
| --- | --- | --- | --- | --- | --- |
| **1-factor-model** | **AIC** | -5631.3 | -2580.3 | -2408.0 | -6185.3 |
|  | **BIC** | -5485.9 | -2434.9 | -2262.5 | -6039.8 |
| **2-factor-model** | **AIC** | -5695.9 | -2615.6 | -2574.4 | -6190.1 |
|  | **BIC** | -5545.6 | -2465.3 | -2424.1 | -6039.8 |

**Supplementary table 2: AIC and BIC values for the 1- and 2-factor-model for the respective neurotransmitter.**

|  | NA | | | |  | DA | | | |  | HT | | | |  | ACh | | | |
| --- | --- | --- | --- | --- | --- | --- | --- | --- | --- | --- | --- | --- | --- | --- | --- | --- | --- | --- | --- |
|  | whole sample  (n = 400) | HC + relatives  (n = 122) | SCD  (n = 152) | MCI/AD  (n = 126) |  | whole sample  (n = 400) | HC + relatives  (n = 122) | SCD  (n = 152) | MCI/AD  (n = 126) |  | whole sample  (n = 400) | HC + relatives  (n = 122) | SCD  (n = 152) | MCI/AD  (n = 126) |  | whole sample  (n = 400) | HC + relatives  (n = 122) | SCD  (n = 152) | MCI/AD  (n = 126) |
| ApoE4 ⟶ high areas | -0.049 | -0.087 | 0.021 | 0.010 |  | -0.033 | -0.108 | -0.034 | 0.109 |  | -0.013 | 0.016 | 0.002 | -0.067 |  | -0.057 | NA | NA | NA |
| age ⟶ high areas | **-0.390***** | **-0.263*** | **-0.363***** | **-0.464***** |  | **-0.388***** | -0.153 | **-0.281**** | **-0.483***** |  | **-0.288***** | **-0.165*** | **-0.485***** | **-0.243*** |  | **-0.355***** | NA | NA | NA |
| education ⟶ high areas | 0.074 | 0.013 | 0.005 | 0.041 |  | 0.030 | 0.147 | -0.137 | -0.079 |  | **0.124**** | 0.127 | 0.014 | 0.094 |  | **0.089*** | NA | NA | NA |
| WMH ⟶ high areas | **-0.114*** | -0.003 | -0.006 | -0.069 |  | 0.084 | **0.356***** | 0.154 | 0.131 |  | **-0.207***** | **-0.205*** | -0.031 | **-0.203*** |  | **-0.147***** | NA | NA | NA |
| gender ⟶ high areas | **-0.300***** | **-0.326***** | **-0.381***** | **-0.370***** |  | **-0.212***** | **-0.343***** | **-0.268**** | -0.066 |  | **-0.315***** | **-0.499***** | **-0.370***** | **-0.310**** |  | **-0.254***** | NA | NA | NA |
|  | | | | |  |  | | | |  |  | | | |  |  | | | |
| ApoE4 ⟶ low areas | 0.018 | -0.044 | 0.013 | 0.151 |  | -0.064 | 0.014 | 0.019 | -0.190 |  | -0.013 | -0.021 | 0.019 | 0.053 |  | 0.038 | NA | NA | NA |
| age ⟶ low areas | **-0.330***** | **-0.196*** | **-0.232*** | **-0.400***** |  | **-0.397***** | **-0.231*** | **-0.440***** | **-0.329**** |  | **-0.471***** | **-0.269*** | **-0.450***** | **-0.558***** |  | **-0.378***** | NA | NA | NA |
| education ⟶ low areas | -0.043 | 0.090 | -0.135 | -0.161 |  | 0.076 | 0.113 | 0.012 | 0.015 |  | 0.041 | 0.166 | -0.093 | -0.088 |  | **0.108*** | NA | NA | NA |
| WMH ⟶ low areas | **0.142**** | **0.320***** | 0.104 | 0.175 |  | **-0.160**** | -0.119 | -0.084 | -0.133 |  | 0.029 | **0.264*** | 0.100 | 0.054 |  | **-0.126*** | NA | NA | NA |
| gender ⟶ low areas | **-0.210***** | **-0.330***** | **-0.312***** | 0.032 |  | **-0.317***** | **-0.520***** | **-0.302***** | **-0.233*** |  | **-0.271***** | **-0.444***** | **-0.431***** | -0.105 |  | **-0.239***** | NA | NA | NA |
|  | | | | |  |  | | | |  |  | | | |  |  | | | |
| ApoE4 ⟶ p-tau | **0.265***** | 0.101 | **0.283***** | **0.203*** |  | **0.265***** | 0.101 | **0.283***** | **0.203*** |  | **0.265***** | 0.101 | **0.283***** | **0.203*** |  | **0.265***** | NA | NA | NA |
| age ⟶ p-tau | **0.271***** | **0.266***** | **0.245**** | **0.194*** |  | **0.271***** | **0.266***** | **0.245**** | **0.194*** |  | **0.271***** | **0.266***** | **0.245**** | **0.194*** |  | **0.271***** | NA | NA | NA |
| education ⟶ p-tau | -0.056 | **0.258**** | -0.066 | -0.080 |  | -0.056 | **0.258**** | -0.066 | -0.080 |  | -0.056 | **0.258**** | -0.066 | -0.080 |  | -0.056 | NA | NA | NA |
| WMH ⟶ p-tau | **0.096*** | **-0.170*** | -0.099 | 0.148 |  | **0.096*** | **-0.170*** | -0.099 | 0.148 |  | **0.096*** | **-0.170*** | -0.099 | 0.148 |  | **0.096*** | NA | NA | NA |
| gender ⟶ p-tau | -0.021 | 0.125 | 0.065 | 0.107 |  | -0.021 | 0.125 | 0.065 | -0.107 |  | -0.021 | 0.125 | 0.065 | -0.107 |  | -0.021 | NA | NA | NA |
|  | | | | |  |  | | | |  |  | | | |  |  | | | |
| ApoE4 ⟶ Aß42/40 | **-0.480***** | **-0.427***** | **-0.479***** | **-0.441***** |  | **-0.480***** | **-0.427***** | **-0.479***** | **-0.441***** |  | **-0.480***** | **-0.427***** | **-0.479***** | **-0.441***** |  | **-0.480***** | NA | NA | NA |
| age ⟶ Aß42/40 | **-0.259***** | -0.091 | **-0.261***** | **-0.247***** |  | **-0.259***** | -0.091 | **-0.261***** | **-0.247***** |  | **-0.259***** | -0.091 | **-0.261***** | **-0.247***** |  | **-0.259***** | NA | NA | NA |
| education ⟶ Aß42/40 | **0.090*** | -0.006 | 0.034 | 0.098 |  | **0.090*** | -0.006 | 0.034 | 0.098 |  | **0.090*** | -0.006 | 0.034 | 0.098 |  | **0.090*** | NA | NA | NA |
| WMH ⟶ Aß42/40 | **-0.123**** | -0.001 | 0.045 | **-0.164*** |  | **-0.123**** | -0.002 | 0.045 | **-0.164*** |  | **-0.123**** | -0.001 | 0.045 | **-0.164*** |  | **-0.123**** | NA | NA | NA |
| gender ⟶ Aß42/40 | -0.008 | -0.104 | -0.019 | 0.052 |  | -0.008 | -0.104 | -0.019 | 0.052 |  | -0.008 | -0.104 | -0.019 | 0.052 |  | -0.008 | NA | NA | NA |
|  | | | | |  |  | | | |  |  | | | |  |  | | | |
| ApoE4 ⟶ memory | -0.021 | -0.062 | 0.045 | -0.152 |  | -0.030 | -0.056 | 0.057 | **-0.169*** |  | -0.041 | -0.073 | 0.045 | **-0.158*** |  | -0.131 | NA | NA | NA |
| age ⟶ memory | -0.016 | 0.028 | **-0.225**** | **-0.160*** |  | -0.044 | -0.008 | **-0.259***** | **-0.164*** |  | -0.038 | 0.025 | **-0.213**** | -0.139 |  | 0.027 | NA | NA | NA |
| education ⟶ memory | **0.195***** | **0.324***** | **0.204**** | **0.247**** |  | **0.217***** | **0.311***** | **0.229***** | **0.258***** |  | **0.191***** | **0.299***** | **0.218***** | **0.262***** |  | **0.166**** | NA | NA | NA |
| WMH ⟶ memory | -0.065 | -0.140 | -0.054 | -0.032 |  | **-0.126**** | **-0.204*** | -0.087 | -0.058 |  | -0.071 | -0.178 | -0.071 | -0.045 |  | -0.084 | NA | NA | NA |
| gender ⟶ memory | 0.070 | -0.099 | **-0.256***** | 0.084 |  | 0.030 | -0.135 | **-0.289***** | 0.048 |  | 0.069 | -0.087 | **-0.251***** | 0.062 |  | 0.051 | NA | NA | NA |
|  | | | | |  |  | | | |  |  | | | |  |  | | | |
| ApoE4 ⟶ language | 0.055 | 0.033 | 0.106 | 0.053 |  | 0.060 | 0.050 | 0.117 | 0.047 |  | 0.049 | 0.032 | 0.102 | 0.055 |  | -0.138 | NA | NA | NA |
| age ⟶ language | -0.063 | -0.028 | **-0.315***** | -0.160 |  | -0.047 | -0.038 | **-0.314***** | -0.147 |  | -0.035 | 0.002 | **-0.265***** | -0.102 |  | 0.017 | NA | NA | NA |
| education ⟶ language | **0.217***** | **0.324***** | **0.206**** | **0.242**** |  | **0.215***** | **0.314***** | **0.215**** | **0.234**** |  | **0.191***** | **0.300***** | **0.210**** | **0.247**** |  | 0.151 | NA | NA | NA |
| WMH ⟶ language | **-0.134**** | **-0.187*** | -0.077 | -0.115 |  | **-0.151***** | **-0.219**** | -0.099 | -0.119 |  | **-0.100*** | **-0.219*** | -0.088 | -0.116 |  | -0.134 | NA | NA | NA |
| gender ⟶ language | 0.073 | -0.101 | **-0.151*** | 0.131 |  | 0.071 | -0.109 | **-0.160*** | 0.136 |  | **0.109**** | -0.068 | -0.112 | 0.145 |  | 0.057 | NA | NA | NA |
|  | | | | |  |  | | | |  |  | | | |  |  | | | |
| ApoE4 ⟶ executive function | 0.086 | 0.139 | 0.117 | 0.086 |  | **0.097*** | 0.155 | 0.124 | 0.088 |  | **0.086*** | 0.140 | 0.113 | 0.093 |  | -0.096 | NA | NA | NA |
| age ⟶ executive function | **-0.135**** | -0.083 | **-0.384***** | -0.167 |  | **-0.100*** | -0.080 | **-0.369***** | -0.151 |  | -0.103 | -0.053 | **-0.341***** | -0.121 |  | -0.057 | NA | NA | NA |
| education ⟶ executive function | **0.175***** | **0.279***** | 0.098 | **0.175*** |  | **0.163***** | **0.272**** | 0.097 | 0.163 |  | **0.142***** | **0.264**** | 0.093 | **0.171*** |  | 0.108 | NA | NA | NA |
| WMH ⟶ executive function | **-0.125**** | **-0.184*** | -0.058 | -0.074 |  | **-0.115**** | **-0.194*** | -0.067 | -0.067 |  | -0.070 | -0.193 | -0.060 | -0.065 |  | -0.116 | NA | NA | NA |
| gender ⟶ executive function | 0.075 | -0.122 | -0.046 | 0.131 |  | **0.093*** | -0.115 | -0.042 | 0.168 |  | **0.121**** | -0.092 | -0.014 | **0.173*** |  | 0.064 | NA | NA | NA |
|  | | | | |  |  | | | |  |  | | | |  |  | | | |
| ApoE4 ⟶ working memory | **0.115*** | 0.122 | **0.167*** | 0.111 |  | **0.130**** | 0.129 | **0.166*** | 0.134 |  | **0.117*** | 0.126 | **0.165*** | 0.129 |  | -0.101 | NA | NA | NA |
| age ⟶ working memory | **-0.111*** | -0.035 | **-0.329***** | -0.157 |  | -0.054 | 0.000 | **-0.277***** | -0.131 |  | -0.079 | -0.002 | **-0.276**** | -0.133 |  | -0.013 | NA | NA | NA |
| education ⟶ working memory | **0.217***** | **0.319***** | 0.123 | **0.216*** |  | **0.199***** | **0.314***** | 0.108 | **0.198*** |  | **0.175***** | **0.311***** | 0.107 | **0.194*** |  | 0.143 | NA | NA | NA |
| WMH ⟶ working memory | **-0.100*** | -0.137 | 0.039 | -0.064 |  | -0.067 | -0.099 | 0.055 | -0.036 |  | -0.020 | -0.086 | 0.053 | -0.030 |  | -0.099 | NA | NA | NA |
| gender ⟶ working memory | **0.105*** | 0.027 | -0.062 | 0.166 |  | **0.148***** | 0.084 | -0.029 | **0.215*** |  | **0.168***** | 0.088 | -0.018 | **0.223*** |  | 0.098 | NA | NA | NA |
|  | | | | |  |  | | | |  |  | | | |  |  | | | |
| ApoE4 ⟶ visual memory | **0.106*** | 0.018 | **0.197*** | 0.090 |  | **0.106*** | 0.018 | **0.194*** | 0.091 |  | **0.098*** | 0.019 | **0.196*** | 0.097 |  | -0.032 | NA | NA | NA |
| age ⟶ visual memory | **-0.161**** | -0.160 | **-0.390***** | **-0.243**** |  | **-0.150**** | -0.159 | **-0.393***** | **-0.246**** |  | **-0.180**** | -0.150 | **-0.366***** | **-0.244**** |  | -0.099 | NA | NA | NA |
| education ⟶ visual memory | **0.207***** | **0.311***** | 0.027 | **0.322***** |  | **0.212***** | **0.317***** | 0.021 | **0.316***** |  | **0.182***** | **0.308***** | 0.019 | **0.312***** |  | **0.170*** | NA | NA | NA |
| WMH ⟶ visual memory | **-0.105*** | **-0.187*** | 0.071 | -0.128 |  | **-0.119**** | -0.175 | 0.077 | -0.122 |  | -0.053 | -0.155 | 0.079 | -0.112 |  | -0.125 | NA | NA | NA |
| gender ⟶ visual memory | **0.106*** | 0.047 | 0.031 | 0.030 |  | **0.108*** | 0.041 | 0.031 | 0.039 |  | **0.136**** | 0.073 | 0.047 | 0.053 |  | 0.097 | NA | NA | NA |

**Supplementary table 3: SEM model results for the covariates of the whole sample models and the multiple group analysis for each neurotransmitter.***** p ≤ 0.001
** p ≤ 0.01
* p ≤ 0.05

**Supplementary figure 1: 2-factor-structural-equation-model for the multiple group comparisons, exemplarily shown for NA.
(a)** with fixed factor loadings across disease groups to test for metric measurement invariance and **(b)** with additional fixation of correlation and regression paths to be equal across groups to investigate intergroup differences in relations between CSF biomarkers, cognitive functions and latent factors ‘areas high in NA’ and ‘areas low in NA’ by testing for structural measurement invariance. First configural invariance has been established by specifying the same model for each of the three investigated groups (HC, SCD and MCI/AD) with free parameter estimation for all regression/correlation paths, variances and residuals. As indicated by the blue arrows in **(a)** the respective factor loadings have been fixed to be equal across the three groups to allow testing metric measurement invariance. After (partial) metric measurement invariance has been found regression paths have been fixed to be equal as shown by the additionally blue colored arrows in **(b)** to compare for differences across groups in the structural part of the model. The different measurement invariance levels have been compared stepwise by ANOVA likelihood-ratio test and restricted paths have been freed up if measurement invariance across groups was not found. This has been done in all neurotransmitter models accordingly.

**Supplementary figure 2: 1- and 2-factor model for the noradrenergic system estimating one or two latent factors for noradrenergic brain areas.**Comparing a 1- to 2-factor-model by separating brain areas into high and low neurotransmitter distribution areas was done for each neurotransmitter (NA, DA, HT, ACh) as shown here exemplarily for NA. **(a)** 1-factor-model with one general latent factor estimated by all included brain area volumes. **(b)** 2-factor-model with two latent factors ‘areas high’ and ‘areas low’ estimated by brain area volumes extracted from high and low neurotransmitter regions, respectively. For all four neurotransmitter Chi-square-difference test showed a better model fit of the data for the 2-factor-model.

After model fit evaluation in the 2-factor-models model fit improvement was performed by checking modification indices. For each neurotransmitter model three intercorrelations between brain area volumes have been added (shown in Fig. 2 and 3). For NA these were intercorrelations between brainstem and cerebellum, brainstem and pallidum, hippocampus and amygdala and fit indices were indicated by CFI: 0.853, TLI: 0.787, RMSEA: 0.132, SRMR: 0.084. For DA intercorrelations between putamen and pallidum, brainstem and pallidum, brainstem and cerebellum were added and fit indices were indicated by CFI: 0.791, TLI: 0.697, RMSEA: 0.130, SRMR: 0.091. For HT intercorrelations between hypothalamus and thalamus, hypothalamus and accumbens, pallidum and putamen were added and fit indices were indicated by CFI: 0.882, TLI: 0.828, RMSEA: 0.110, SRMR: 0.068. For ACh intercorrelations between brainstem and hypothalamus, brainstem and pallidum, pallidum and putamen were added and fit indices were indicated by CFI: 0.845, TLI: 0.774, RMSEA: 0.141, SRMR: 0.082.

**Supplementary figure 3: Correlation matrices of all included brain area volumes averaged across hemispheres and corrected by total intracranial volume for each neurotransmitter in the whole sample (n = 400).
(a)** NA: highest correlations were observed for amygdala and hippocampus and accumbens and amygdala.
**(b)** DA: highest correlations were observed for cingulate cortex and frontal cortex, brainstem and cerebellum and occipital cortex and frontal cortex.
**(c)** HT: highest correlations were observed for parietal cortex and temporal cortex, frontal cortex and temporal cortex, frontal cortex and parietal cortex and insula and temporal cortex.
**(d)** ACh: highest correlations were observed for hippocampus and amygdala, temporal cortex and amygdala, temporal cortex and hippocampus and accumbens and amygdala.
Crosses indicating non-significant correlations (p-value > 0.1).
